# Antioxidant hepatic lipid metabolism can be promoted by orally administered inorganic nanoparticles

**DOI:** 10.1038/s41467-023-39423-3

**Published:** 2023-06-20

**Authors:** Jie Cai, Jie Peng, Juan Feng, Ruocheng Li, Peng Ren, Xinwei Zang, Zezong Wu, Yi Lu, Lin Luo, Zhenzhen Hu, Jiaying Wang, Xiaomeng Dai, Peng Zhao, Juan Wang, Mi Yan, Jianxin Liu, Renren Deng, Diming Wang

**Affiliations:** 1 College of Animal Sciences, Dairy Science Institute, MOE Key Laboratory of Molecular Animal Nutrition, Zhejiang University, Hangzhou, 310029 PR China; 2 Department of Veterinary Medicine, College of Animal Sciences, Zhejiang University, Hangzhou, 310029 PR China; 3 State Key Laboratory of Silicon and Advanced Semiconductor Materials, Institute for Composites Science Innovation, School of Materials Science and Engineering, Zhejiang University, Hangzhou, 310027 China; 4 Institute of Environmental Health, MOE Key Laboratory of Environmental Remediation and Ecosystem Health, College of Environmental & Resource Sciences, Zhejiang University, Hangzhou, 310058 PR China; 5 Department of Medical Oncology, The First Affiliated Hospital, School of Medicine, Zhejiang University, Hangzhou, 310003 PR China

**Keywords:** Cell-particle interactions, Nanoparticles, Nanomedicine

## Abstract

Accumulation of inorganic nanoparticles in living organisms can cause an increase in cellular reactive oxygen species (ROS) in a dose-dependent manner. Low doses of nanoparticles have shown possibilities to induce moderate ROS increases and lead to adaptive responses of biological systems, but beneficial effects of such responses on metabolic health remain elusive. Here, we report that repeated oral administrations of various inorganic nanoparticles, including TiO_2_, Au, and NaYF_4_ nanoparticles at low doses, can promote lipid degradation and alleviate steatosis in the liver of male mice. We show that low-level uptake of nanoparticles evokes an unusual antioxidant response in hepatocytes by promoting *Ces2h* expression and consequently enhancing ester hydrolysis. This process can be implemented to treat specific hepatic metabolic disorders, such as fatty liver in both genetic and high-fat-diet obese mice without causing observed adverse effects. Our results demonstrate that low-dose nanoparticle administration may serve as a promising treatment for metabolic regulation.

## Abstract (web summary)

Inorganic nanoparticles can accumulate in living organisms causing a dose-dependent increase in cellular reactive oxygen species. Here, the authors leverage this as a potential scheme for regulating metabolic disorders, showing that that low-dose nanoparticle administration can enhance lipid hydrolysis and alleviate fatty liver.

## Introduction

Inorganic nanoparticles have emerged as promising functional building blocks for various applications in biomedicine, but our understanding of their interactions with biological systems in vivo remains inadequate^1–5^. It becomes increasingly accepted that endocytosis of inorganic nanoparticles, such as metal, silica, metal-oxide, and lanthanide-based nanoparticles, results in elevated intracellular reactive oxygen species (ROS) generation^6–10^. Consequently, the increased ROS supply shifts the balance between the production of ROS and antioxidant defense in a biological system. Many studies to date have suggested that high doses of nanoparticles generate considerable toxicity in living organisms as they cause imbalance of cellular ROS response which leads to a series of pathological events such as oxidative stress, apoptosis, inflammation, fibrosis, and carcinogenesis^11–17^. However, very few work has systematically investigated the influence of low-dose nanoparticles on ROS-related metabolic regulations^18–22^. It remains uncertain how nanoparticles interact with molecular cascade involving low-level ROS response and the downstream antioxidant biological processes.

Liver is a metabolic center for collecting, metabolizing, and transporting substances in living bodies^23,24^. Due to the worldwide increase of overweight and obesity, aberrant liver lipid metabolism which leads to diseases such as non-alcoholic fatty liver and liver damages has become a prevalent health threat^25–28^. However, the diagnosis and treatment of aberrant liver lipid metabolism is difficult; and there is a lack of effective treatments and clinical drugs at present^29,30^. Liver is known as one of the key organs responsible for nanoparticle capture and clearance assisted by its reticuloendothelial system^31^. Liver sequestration is a barrier to translating nanomedicines, preventing the delivery of nanoparticles to desired targets. Nonetheless, such a disadvantageous characteristic of preferential liver uptake opens up potential opportunities for targeted hepatic intervention with nanoparticles.

**Fig. 1:**
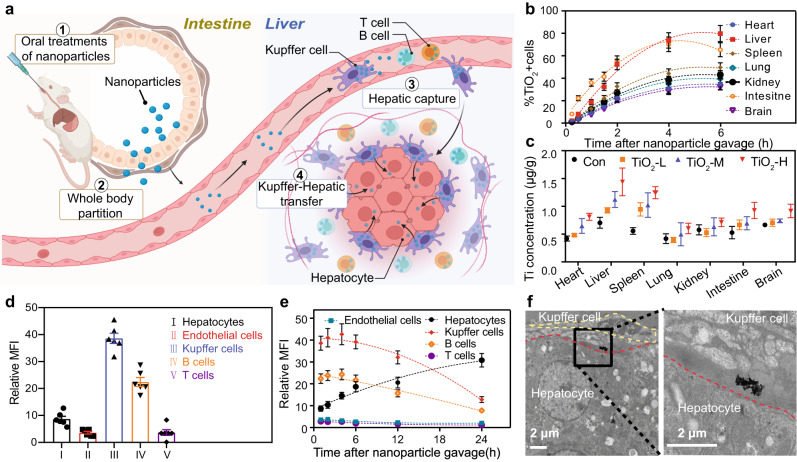
In vivo transfer of orally administrated nanoparticles and their targeted liver accumulation. **a** Schematic illustration (Created with BioRender.com) showing in vivo delivery of orally administrated nanoparticles to liver tissues. **b** Time-dependent distribution of nanoparticle-positive cells in the main organs of wild-type (WT) mice after treatment with gavage of fluorescence (Cy5.5)-modified TiO_2_ nanoparticles (0.72 mg/kg). The detailed gating strategy is provided in supplementary information, Fig. S6. **c** Elemental analysis showing the bio-distributions of Ti elements in the main organs of mice 21 days after nanoparticle gavage. TiO_2_-L, TiO_2_-M, and TiO_2_-H represent mice groups treated with low-dose (0.72 mg/kg/day), middle-dose (1.8 mg/kg/day), and high-dose (18 mg/kg/day) of TiO_2_ nanoparticles, respectively. **d** Relative mean fluorescence intensity (MFI) of different types of liver cells indicating the accumulation of Cy5.5-modified TiO_2_ nanoparticles in different liver cells in WT mice 1 h after gavage of Cy5.5-modified TiO_2_ nanoparticles (1.8 mg/kg). **e** Relative MFI changes in different types of liver cells indicating dynamic changes of nanoparticle distributions in liver cells as a function of time after Cy5.5-modified TiO_2_ nanoparticle gavage. **f** Representative transmission electron microscopy (TEM) images showing the Kupffer-hepatocyte nanoparticle transfer. The TEM samples were collected 6 h after gavage of nanoparticles. Data in (**b**–**e**) are presented as mean values ± SEM. *n* = 6. Source data are provided as a Source Data file.

Here, we report the alleviation of lipid metabolism disorders in liver by using orally administrated non-cytotoxic inorganic nanoparticles. We demonstrate that a variety of small (sub-20 nm) inorganic nanoparticles gain access to hepatocytes after gastrointestinal absorption (Fig. 1a). More importantly, we show that repeated oral administrations of a very low dose (<1 mg/kg/day) of solid nanoparticles trigger a low-level antioxidant defense against nanoparticle-induced ROS by *Ces2h* gene-mediated lipid metabolism in hepatocytes. This unconventional antioxidant response promotes hydrolysis of esters, leading to a decrease in lipid accumulation and alleviation of steatosis in the liver, without significant side effects. Our results indicate that non-cytotoxic inorganic nanoparticles may act as promising agents for the prevention and treatment of obesity and non-alcoholic fatty liver diseases.

## Results

### Transportation kinetics of orally administrated inorganic nanoparticles in living mice

Three types of inorganic nanoparticles (TiO_2_, NaYF_4_, and Au nanoparticles) were employed as model nanodrugs for investigation, owing to the low immediate cytotoxicity and degradability of these nanoparticles in biological systems^32–34^. Small-sized nanoparticles (~14–17 nm) were adopted to ensure optimized in vivo targeting towards hepatic organisms (Fig. S1–S4). We first performed single-dose oral administration of nanoparticles at 0.72 mg/kg in wild-type (WT) mice to characterize the in vivo transportation and biodistribution of these nanoparticles. After gavage administration, TiO_2_ nanoparticles were found to penetrated into the enterocytes without damaging intestinal integrity (Fig. S5a). Flow cytometry combined with intensive sampling was carried out next to monitor the post-administration partition of TiO_2_ nanoparticles in the whole body. It was found that all the three inorganic nanoparticles were first absorbed by the intestine 4 h post-administration, followed by simultaneous accumulation in various organs, including the liver, spleen, lung, and kidney, through both blood and lymph circulations (Fig. 1b; Fig. S5b). Nonetheless, we observed that the liver acquired apparently higher amounts of nanoparticles, suggesting higher ability in capturing nanoaprticles by the hepatic system. This was also supported by a long-term gavage study (0.72, 1.8, and 18 mg/kg/day for 21 days), in which liver was found to present highest accumulation (*P* < 0.05) of TiO_2_ nanoparticles across all the tested organs (Fig. 1c). We observed similar trends for Au and NaYF_4_ nanoparticles, regardless of surface chemistry (Fig. S5d, e). These findings suggested that it was possible to target inorganic nanodrugs selectively towards hepatic cells through oral administration^35,36^.

**Fig. 2:**
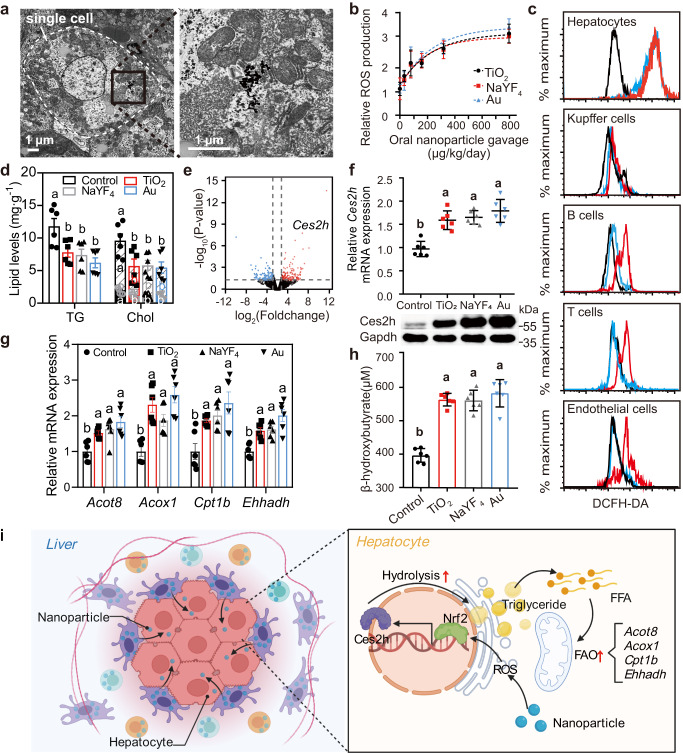
Promotion of hepatic lipid metabolism by nanoparticle-activated *Ces2h* expression. **a** Transmission electron microscopy images indicating the subcellular distributions of TiO_2_ nanoparticles in a hepatocyte 24 h after nanoparticle gavage (0.72 mg/kg). **b** Nanoparticle-dose-dependent hepatocyte ROS production by different nanoparticles at 18 h after nanoparticle gavage. **c** Flow cytometry showing ROS generation in different types of liver cells. The black, blue, and red curves represent liver samples collected at 6, 18, 24 h after TiO_2_ nanoparticle gavage (0.72 mg/kg), respectively. **d** Effect of different types of nanoparticle treatments (0.72 mg/kg/day for 21 days) on liver lipid levels in wild-type (WT) mice. TG triglyceride, Chol cholesterol. The full length of the column represents the total cholesterol level (significance indicated above the column), the color-filled part represents the cholesterol ester level (significance indicated inside the column, in white), and the diagonally-filled part represents the free cholesterol level (significance indicated inside the column, in corresponding color). **e** Volcano plot of liver transcriptomic comparison between the TiO_2_-treated group (0.72 mg/kg) and the control group of WT mice (*n* = 5). FoldChange = TPM_TiO2_/TPM_Control_. **f** Relative hepatic *Ces2h* mRNA expressions measured by RT-qPCR (upper), and Ces2h protein expression measured by western blotting (lower) in WT mice. **g** Hepatic mRNA levels of fatty acid oxidation (FAO) genes in mice measured by RT-qPCR. **h** Plasma β-hydroxybutyrate levels in WT mice. **i** Schematic diagram (Created with BioRender.com) showing the reduction of liver triglycerides by nanoparticles. Different letters indicate the significant difference (*P* < 0.05) analyzed by one-way ANOVA. Expect for otherwise specified, data in (**b**, **d**, **f–h**) are presented as mean values ± SEM. *n* = 6. Source data are provided as a Source Data file.

To understand the biokinetics of inorganic nanoparticles in the liver, we performed a flow cytometry method to monitor the time-dependent acquisition of TiO_2_ nanoparticles in different types of liver cells (Fig. S6; Fig. S7). Figure 1d indicated the biodistribution of TiO_2_ nanoparticles in various liver cells 1 h post-administration. As can be seen, the key contributors for nanoparticle uptake were Kupffer cells with the highest relative mean fluorescence intensity (MFI) of 38.6, followed by B cells with a relative MFI of 22.4. Endothelial cells showed weakest acquisition, despite their close contact with the circulatory system. Interestingly, we inferred a shifting of nanoparticles from Kupffer and B cells to hepatocytes 24 h post-administration, as we observed a substantial decrease in relative MFI of Kupffer and B cells and a concurrent increase in relative MFI of hepatocytes (Fig. 1e). A representative transmission electron microscope (TEM) image also indicated the persistence of nanoparticles in the cellular junctions between Kupffer cell and hepatocyte 6 h after gavage of nanoaprticles, supporting the transportation of nanoparticles between these two cell types (Fig. 1f). This observation agreed with recent reports where Kupffer-hepatocyte transfers were noticed in low-density lipoprotein nanoparticles, which is a kind of endogenous nutrient nanoparticles with approximate diameter of 25.5 nm^37^. Moreover, we found that the transferred nanoparticles were mostly localized in the endoplasmic reticulum (ER)-mitochondria system within hepatocytes (Fig. 2a). This finding accounted for the longtime retention of nanoparticles in hepatocytes as substances delivered into mitochondria were more difficult to efflux than those distributed in the cytoplasm^38^.

### Nanoparticle-mediated cascade effects on Nrf2-*Ces2h* lipid metabolism in liver

As both the ER and mitochondria are abundant sources and targets related to in vivo ROS generation^39,40^, we investigated the liver ROS production influenced by inorganic nanoparticles. After gavage of TiO_2_ nanoparticles (0.72 mg/kg) for 24 h, nanoparticles did not lead to significant changes in ROS production at the whole liver level (*P* = 0.09; Fig. S8a). Nevertheless, we noticed a substantially higher level of ROS production in hepatocytes than in other liver cell types (*P* < 0.01; Fig. 2b, c; Fig. S8b). With an increasing nanoparticle dosage (from 0 to 800 μg/kg), a dose-dependent ROS production was detected in a form of Boltzmann sigmoidal increased manner within the hepatocytes (Fig. 2b). The specific ROS generation in hepatocytes agreed with the observation that most of the nanoparticles accumulated in hepatocytes in 24 h post-administration, suggesting liver cellular heterogeneity in response to TiO_2_ inorganic nanoparticles. Since hepatocytes possess high mitochondrial content and express biological oxidation genes, the high ROS expression may contribute to the abundance of ER-mitochondria systems in hepatocytes^41^. Additionally, the hepatic ROS generation by orally administrated nanoparticles was also applied to NaYF_4_ and Au nanoparticles (Fig. S8b). It revealed that endocytic inorganic nanoaprticles can promote ROS production in selective liver cells.

We next examined whether the promoted ROS generation was sufficient to affect the hepatic signaling pathway during long-term nanoparticle administration. We orally treated groups of WT mice with TiO_2_, NaYF_4_, and Au nanoparticles, respectively, at the same dosages of 0.72 mg/kg/day for 21 days. After the treatment, we observed a pronounced elevation of nuclear factor erythroid 2-related factor 2 (Nrf2; a critical in vivo antioxidant responder) in liver (Fig. S9a–c)^42^. Nrf2 is a cellular transcription factor responsible for preventing increased levels of oxidative stress upon nanoparticle exposure^43,44^. It was found that the stimulated Nrf2 signals were mainly localized to hepatocytes, demonstrating the correlation of oral nanoparticles in regulating Nrf2 expression. More intriguingly, the elevated Nrf2 by nanoparticles seemed qualified to reprogram the hepatic lipid metabolism as oral nanoparticles reduced hepatic triglycerides and cholesterol levels (including free cholesterol, cholesterol ester, and total cholesterol) by 34%-47% without causing significant body weight changes of WT mice (Fig. 2d; Fig. S10a). However, oral nanoparticle treatments failed to alter the hepatic lipid metabolism in Nrf2-deficient (*Nrf2*^*–/–*^) mice, again confirming the roles of nanoparticles in triggering Nrf2 expression and Nrf2-dependent hepatic lipid reprogramming (Fig. S9i–j; Fig. S11).

We then carried out transcriptome analysis to screen the downstream targets of Nrf2 corresponding to the promoted lipid metabolism. The results showed that after nanoparticle administration, *Ces2h* that encodes carboxylesterase 2 enzyme in catalyzing ester hydrolysis, was highly expressed in livers of WT mice (Fig. 2e, f; Fig. S10b). Of note, while carboxylesterase 2 gene family has eight members including *Ces2a* to *Ces2h*, only *Ces2h* was significantly increased by nanoparticle treatments (Fig. S10c). The genome prediction also suggested that *Ces2h* expression can be regulated by Nrf2 signaling pathway, as the binding element (antioxidant response element; ARE) of Nrf2 is located at promoters of both mouse (*Ces2h*) and human (*CES2*) Ces2 genes (Fig. S9d). Moreover, we observed that the nanoparticle treatments did not significantly promote the *Ces2h* mRNA and its encoded Ces2h protein expression in *Nrf2*^*–/–*^ mice, confirming that Nrf2 is essential in *Ces2h* up-regulation (Fig. S9i, m). We also found that oral administration of inorganic nanoparticles promoted the binding of Nrf2 to *Ces2h* proximal promoter and alleviated *Ces2h* expression in *Nrf2*^*+/+*^ WT mice. As evidence, chromatin immunoprecipitation (ChIP) analysis showed that the nanoparticle treatment increased the binding of Nrf2 to a proximal *Ces2h* promoter containing ARE (*Ces2h* ARE s1), but not to a non-specific region (*Nqo1* NS) 2 kilobases away from this ARE (Fig. S9n). We further performed a Nrf2-*Ces2h* cascade analysis by an already known small molecule Nrf2 activator, bardoxolone (Fig. S9e–g). The Nrf2 protein expression was elevated by bardoxolone treatment, accompanied with its known downstream target *Nqo1* transcripts. In parallel, *Ces2h* expression was also elevated by bardoxolone treatment in WT mice, but not in *Nrf2*^*-/-*^ mice (Fig. S9g; Fig. S9k), which further confirmed *Ces2h* as a downstream target of Nrf2. Taken together, these results demonstrated that Nrf2 elevation and *Ces2h* activation represented a direct upstream and downstream signaling cascade effect.

The Nrf2-*Ces2h* cascade effect was found to reduce hepatic lipid levels by promoting possible ester hydrolysis according to the function of the family Ces2h belongs to^45–47^, and subsequent fatty acid oxidation (FAO). Compared to the untreated groups, we observed that plasma β‐hydroxybutyrate levels in nanoparticle-treated mice increased from ~400 to ~550 μM, accompanied by enhanced expressions of several FAO-related genes including *Acot8*, *Acox1*, *Cpt1b*, and *Ehhadh* (Fig. 2g, h). On the other hand, we demonstrated that oral nanoparticles displayed little effect on very low-density lipoprotein (VLDL) secretion, as well as on the expression of hepatic apolipoprotein B (*Apob*) and microsomal triglyceride transfer protein (*Mttp*) in chow-fed WT mice (Fig. S12a, c). Besides, oral nanoparticle treatments were found to have negligible effect on hepatic de novo lipogenesis as evidenced by ^2^H_2_O isotope labeling examination of newly synthesized [^2^H]palmitate and [^2^H]cholesterol and lipogenic gene or re-esterification gene screening (Fig. S12b, d, e, f). These findings indicate that nanoparticles only promote possible ester hydrolysis and FAO without significant side effects on lipogenesis, re-esterification, or lipid secretion (Fig. 2i). However, possible ester hydrolysis and subsequent FAO were not affected by nanoparticle treatment under conditions in absent of Nrf2 or *Ces2h* (Fig. S11; Fig. S13; Fig. S14). It suggested that the metabolic pathway had a direct nanoparticle-ROS-Nrf2-*Ces2h* signaling cascade in eliminating hepatic lipids.

### Antioxidant responses of nanoparticle-*Ces2h* cascades

As Nrf2 mainly mediates transcription of genes encoding antioxidant and detoxification enzymes to prevent increased levels of cellular oxidative stress^48,49^, we wondered why the *Ces2h*-hepatic lipolysis pathway was activated by Nrf2 as the ROS level of hepatocytes increased. To this regard, we generated *Ces2h*-deficient (*Ces2h*^*–/–*^) mice. The hepatic mRNA expressions of *Ces2h* and protein levels of Ces2h were much lower in *Ces2h*^*–/–*^ mice than in WT mice (Fig. S10f, g). The knockout efficiency was specific for *Ces2h* in either liver or intestine and had no significant influence on other *Ces2* genes (*Ces2a-g*), triglyceride lipase gene (*Atgl*), or re-esterification genes (*Dgat1*, *Dgat2*, *Gpat1*, *Gpat2*) (Fig. S10f, h). As *Ces2h* is a member of Ces2 family and a potentially important triglyceride lipase, the *Ces2h*^*-/-*^ mice exhibited steatosis (Fig. S10i). We then treated *Ces2h*^*–/–*^ mice with inorganic nanoparticles to investigate the role of *Ces2h* in responding to nanoparticle-induced ROS and Nrf2. In contrast to WT mice, the nanoparticle treatments did not change the body weight, liver weight, or hepatocyte triglyceride levels of *Ces2h*^*–/–*^ mice (Fig. S10j–k). Loss-of-function experiments revealed that *Ces2h*^*–/–*^ hepatocytes exhibited higher ROS levels than WT ones (Fig. S15a, b). The nanoparticle treatments (40 days oral administration) were found to further increase cellular ROS levels of all mice compared to the untreated counterparts. We also observed that the nanoparticles did not threaten to cell viability (89–91%) for normal hepatocytes, while the survival rate of *Ces2h*^*–/–*^ hepatocytes significantly decreased (77–82%). This finding suggested that the inorganic nanoparticles were more cytotoxic to *Ces2h*^*–/–*^ hepatocytes than normal hepatocytes. Accordingly, in vitro cell viability analysis revealed that the inorganic nanoparticles were more cytotoxic to *Ces2h*^*–/–*^ hepatocytes, as they suffered from a higher mortality rate compared to normal *Ces2h*^*+/+*^ cells (Fig. S15b). More seriously, while all the normal WT mice survived during 40 days of nanoparticle treatment, the death rate increased in *Ces2h*^*–/–*^ mice treated with the same dosage of nanoparticles (Fig. S10l). In contrast to WT mice, serum liver indexes including alanine aminotransferase (ALT) and aspartate aminotransferase (AST) were significantly increased (Au) or tended to increase (TiO_2_, and NaYF_4_) in *Ces2h*^*-/-*^ mice after the treatments, indicating slight liver damage in these mice (Fig. S10i, m, n). The results strongly suggested that besides possibly catalyzing ester hydrolysis, Ces2h possessed an additional function of physiologically controlling the hepatocyte ROS level, despite the fact that such a function has never previously been recognized.

**Fig. 3:**
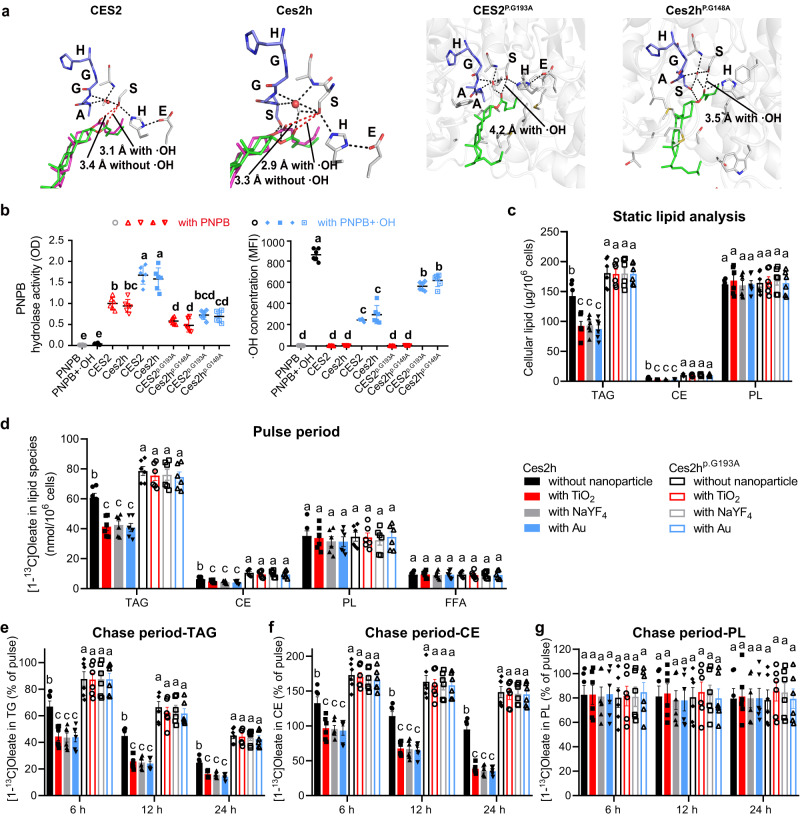
Carboxylesterase 2 (CES2/Ces2) antioxidant response to ROS in the presence of nanoparticles. **a** Computational simulations of molecular docking of normal CES2/Ces2h or mutated CES2^p.G193A^/Ces2h^p.G148A^ with cholesterol ester and ·OH. Blue stick: oxyanion hole; Green sticks: spatial configuration of ligands with·OH in the oxyan hole; Pink sticks: spatial configuration of ligands without ·OH in the oxyan hole; Black dashes: hydrogen-bond interactions. **b** In vitro catalytic activity and corresponding ·OH concentration changes of normal CES2/Ces2h or mutated CES2^p.G193A^/Ces2h^p.G148A^ enzymes on catalyzing PNPB hydrolysis under different conditions. **c** Intracellular lipid fraction analysis of normal Ces2h or mutated Ces2h^p.G148A^ hepatocyte cell lines upon treatments with TiO_2_, NaYF_4_, and Au nanoparticles, respectively. **d** Incorporation of [1-^13^C]oleate into cellular lipids. **e–g** Chase experiments evaluating the turnover of lipid species, including triglycerides (TG), cholesterol esters (CE), and phospholipids (PL). Statistics for the chase period were analyzed as a percentage of the pulse. The group setting was the same in (**c–g**) as indicated in (**d**). Different letters indicate the significant difference (*P* < 0.05) analyzed by one-way ANOVA. Data in (**b**–**g**) are presented as mean values ± SEM. *n* = 6. Source data are provided as a Source Data file.

To understand the underlying catalytic processes and identify ROS consumption pathways, we performed homologous modeling and docking analysis of *Homo sapiens* and *Mus musculus* carboxylesterase 2 (CES2/Ces2h) enzymes. Both the CES2 and Ces2h enzymes contain an oxyanion hole adjacent to the catalytic triad which is typically occupied by a H_2_O molecule. The filled H_2_O forms a hydrogen bonding network with glycine-alanine or glycine-serine (GA/GS) residues of the oxyanion hole or the serine residue from the serine-histidine-glutamic acid catalytic triad (Fig. S17). We noticed that the H_2_O molecule can be replaced by hydroxyl radical (·OH; a main cellular ROS) forming stronger hydrogen bonds with the surrounding GA/GS residues (Figs. S18, S19). More importantly, when the enzymes approach cholesterol ester, the new hydrogen bonds between the hydroxyl on the catalytic serine and the oxygen atom on the ester group are shorter than the original ones (Fig. 3a). It was inferred that the interaction and catalytic capacity of CES2/Ces2h enzymes may improve when ·OH is introduced. To further confirm the role of ·OH, we generated mutated CES2 (CES2^p.G193A^) and Ces2h (Ces2h^p.G148A^) having abnormal compressed oxyanion holes. The modeling results indicated that the mutation prevented H_2_O from entering into the oxyanion holes and lengthened the hydrogen bonds between the enzyme catalytic triad and the cholesterol ester group, which would probably reduce catalytic activity of mutated enzymes (Fig. 3a).

We next carried out in vitro enzyme catalysis tests to experimentally validate the consumption of ·OH by CES2/Ces2h enzymes. We measured the reaction activity and residue ·OH concentration of CES2, CES2^p.G193A^, Ces2h, and Ces2h^p.G148A^ for catalyzing an ester hydrolase substrate, namely p-nitrophenyl butyrate (PNPB). The in vitro catalysis experiment showed that introduction of ·OH facilitated esterase activity of both CES2 and Ces2h (Fig. 3b, Fig. S21–S24). Additionally, the residue ·OH in all the reaction groups decreased compared to the PNPB + ·OH control group, which did not add any enzyme, confirming that ·OH can be consumed by CES2/Ces2h during the hydrolase reaction (Fig. 3b).

The ability of inorganic nanoparticles to facilitate ester hydrolysis in living cells was further verified by analyzing intracellular lipid fractions and lipid turnover (Fig. 3**;** Fig. S20). Mutated CES2^p.G193A^/Ces2h^p.G148A^ hepatocytes had higher levels of triglycerides and cholesterol esters but similar levels of phospholipids, compared to their normal counterparts, indicating that CES2/Ces2h can hydrolyze triglycerides and cholesterol esters, thus influencing subsequent turnover. Normal CES2/Ces2h hepatocytes with nanoparticle treatments exhibited lower levels of triglycerides and cholesterol esters, but not phospholipids, than those without nanoparticle treatments, while mutated CES2^p.G193A^/Ces2h^p.G148A^ hepatocytes with or without nanoparticle treatments showed no significant differences in levels of triglycerides and cholesterol esters. These findings supported the notion that nanoparticles can enhance the hydrolysis of triglycerides and cholesterol esters, and subsequent lipid turnover in hepatocytes by promoting CES2/Ces2h-mediated ester hydrolysis.

### Regulation of lipid metabolism disorders by orally ingested inorganic nanoaprticles

**Fig. 4:**
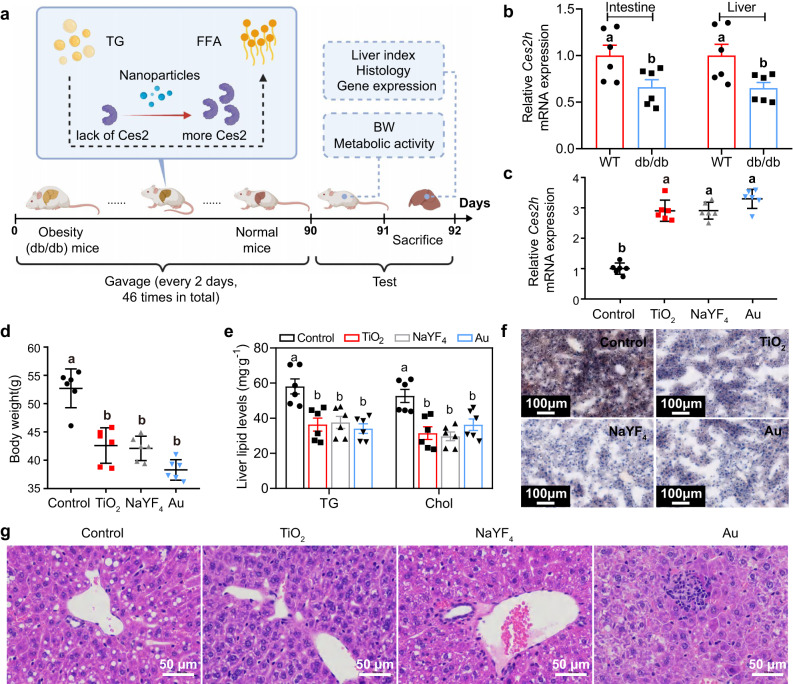
Nanoparticle administration for treatment of lipid metabolic disorder in genetic obesity (*db/db*) mice by restoration of hepatic *Ces2h* expression. **a** Scheme (Created with BioRender.com) showing the oral administration procedure for nanoparticle treatments (0.72 mg/kg per dose) over 92 days on *db/db* mice. FFA, free fatty acids. **b** Relative *Ces2h* mRNA expression, measured by RT-qPCR, in enterohepatic systems of wild-type (WT) or *db/db* mice fed with chow food in the absence of nanoparticle treatment. **c** Relative *Ces2h* mRNA expression, measured by RT-qPCR, in the liver of *db/db* mice on day 92 of nanoparticle treatments with different types of nanoparticles. **d** Body weight of *db/db* mice on day 92 of nanoparticle treatments with different types of nanoparticles. **e** Triglyceride (TG) and total cholesterol (Chol) contents in liver of *db/db* mice after nanoparticle treatments. **f** Oil-red O and (**g**), Hematoxylin-eosin (HE) staining of liver tissues of *db/db* mice collected on day 92 of the treatments. Different letters indicate the significant difference (*P* < 0.05) analyzed by one-way ANOVA. Data in (**b**–**e**) are presented as mean values ± SEM. *n* = 6. Source data are provided as a Source Data file.

Given the ability of promoting hepatic lipolysis, orally ingested inorganic nanoparticles are potentially useful for treating obesity caused by lipid metabolism disorders. As a proof-of-concept experiment, we orally treated three groups of genetically obese *db/db* mice with TiO_2_, NaYF_4_, and Au nanoparticles (0.72 mg/kg every 2 days for three months), respectively (Fig. 4a). Before treatment, the *db/db* mice showed liver steatosis with 65.5 ± 7.1% lower intestinal and hepatic *Ces2h* mRNA levels compared to WT mice (Fig. 4b). After treatment, the relative *Ces2h* mRNA expression of *db/db* mice increased by ~2.9–3.3 times, depending on the types of nanoparticles (Fig. 4c). In all treated groups, the nanoparticle treatments were observed to cause over 20% body weight reduction along with ~31.1-43.6% decrease in hepatic triglyceride and cholesterol concentration levels (Fig. 4d, e). Oil-red O and Hematoxylin-eosin (HE) staining of liver tissues revealed that nanoparticle treatments reduced neutral lipid accumulation and alleviated hepatic steatosis (Fig. 4f, g).

**Fig. 5:**
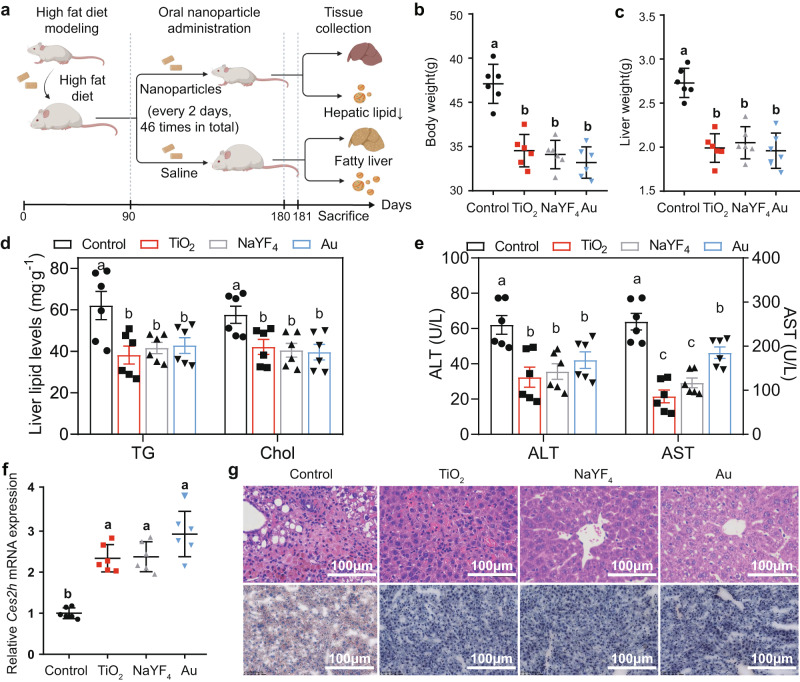
Improvement of lipid metabolism by nanoparticle administration in C57BL/6 wild-type (WT) mice fed a high fat diet (HFD). **a** Scheme showing the oral administration procedure for nanoparticle treatments over 91 days on WT mice fed a HFD for 90 days. The oral treatment of nanoparticles was performed by a 3-month gavage (0.72 mg/kg per dose). Body (**b**) and liver (**c**) weight, hepatic triglyceride (TG) and total cholesterol (Chol) levels (**d**), Plasma alanine aminotransferase (ALT) and aspartate aminotransferase (AST) levels (**e**), and relative *Ces2h* mRNA expression, measured by RT-qPCR, (**f**) of HFD-fed C57BL/6 mice on day 181 of nanoparticle treatments. **g** Hematoxylin-eosin (HE) and Oil-red O staining of liver tissues of WT mice collected on day 181 of the treatments. Different letters indicate the significant difference (*P* < 0.05) analyzed by one-way ANOVA. Data in (**b**–**f**) are presented as mean values ± SEM. *n* = 6. Source data are provided as a Source Data file.

Ingested inorganic nanoparticles can regulate genetic obesity and alleviate obesity and fatty liver diseases caused by high fat diet (HFD). Our experiments showed that administration of inorganic nanoparticles for 3 months resulted in significant body and liver weight loss in HFD-fed mice owing to a reduction in hepatic triglyceride and cholesterol levels by elevating hepatic *Ces2h* expression (Fig. 5a–d, f). Notably, nanoparticle treatments did not alter metabolic activities of HFD-fed mice, such as food intake, O_2_ consumption, and CO_2_ production (Fig. S25). In all treated HFD-fed mice groups, the fatty liver-induced liver damage was relieved, as we observed reduction of plasma ALT and AST levels, and recovery of liver tissue morphology after treatments (Fig. 5e, g).

The genes related to VLDL triglyceride secretion or hepatic de novo lipogenesis were not changed by oral nanoparticle treatments in both HFD-fed and *db/db* mouse models (Fig. S26a–d). Nonetheless, the expression of peroxisome proliferator‐activated receptor alpha (PPAR‐α), a nuclear receptor that regulates FAO, was enhanced (Fig. S26e). PPAR‐α can bind with free fatty acid (FFA) and function as a transcription factor to initiate FAO. Since FFA was highly produced via Ces2h-mediated lipolysis, the expression of PPAR‐α target genes, including *Cpt1b*, *Ehhadh*, *Acox1*, and *Acot8*, increased significantly after nanoparticle treatments (Fig. S26f, g). Consequently, plasma β‐hydroxybutyrate, an indicator of hepatic FAO, increased in both *db/db* and HFD‐fed mice after nanoparticle treatments (Fig. S26h, i). The results in both mouse models clearly demonstrated that ingested inorganic nanoparticles were capable of remodeling hepatic lipid metabolism by improving ester hydrolysis in animals with fatty liver (Fig. S26j).

### Biological safety assessment of orally administrated nanoparticles

Despite previous studies have shown that nanoparticles may cause a range of toxicity at high doses (e.g. oral treatment with >50 ~ 5000 mg/kg of TiO_2_ nanoparticles)^50–52^, the low-dose oral nanoparticle administration in our treatment was found not to generate any observed adverse effects. HE staining and hematology analysis of *db/db* and HFD-fed mice showed that the 3-month nanoparticle treatments did not induce tissue injury, inflammation, or oxidative stress in major organs (Fig. S27). Although we observed considerable nanoparticle accumulation in the gastrointestinal system, including liver, spleen, and intestine, these nanoparticles displayed complete clearance in all tested organs within 21 days post-treatments (Fig. S27a-b). These findings suggested that orally administrated nanoparticles have fast clearance and low toxicity. In contrast, it was reported that airway or intravenous administration of nanoparticles showed slower clearance (up to 30 days) and higher toxicity^53–56^.

The trigger dose for the nanoparticle-*Ces2h* response is actually much lower than the upper dose limit for safe use of these nanoparticles. For example, when the dose was increased to 25 times (18 mg/kg/day for 21 days) higher than the trigger dose (0.72 mg/kg/day for 21 days), no observed retention or pathological change was found for WT-mice with normal Ces2h responses (Fig. S28–33). Nevertheless, even low doses (0.72 mg/kg/day for 21 days) of nanoparticles in *Ces2h*^*-/-*^ mice led to abnormal signal amplification due to the absence of effectors. We identified classical biological processes related to nanotoxicity, including oxidative stress and inflammation, in *Ces2h*^*−/−*^ mice treated with nanoparticles (Figs. S30, S31). These results further confirmed that Ces2h was able to perform lipid hydrolysis while maintaining normal physiological levels of ROS, without causing tissue damage.

## Discussion

Roles of carboxylesterases in lipid metabolism and homeostasis have been revealed by genetic and chemical/drug manipulations in mice or cells^57^. Although recent advances demonstrated the relevance of carboxylesterases, mainly the CES1/Ces1 and CES2/Ces2 families, to metabolic diseases such as obesity and fatty liver disease, differences and similarities among carboxylesterases were elucidated. Expressions of CES1/Ces1 family are positively related to fatty liver disease and obesity^58^. In contrast, hepatic CES2/Ces2 expressions are considered to prevent fatty liver disease by improving glucose tolerance and insulin sensitivity^45^. Thus, the specific restoration of liver CES2/Ces2 expression, but not CES1/Ces1, can be a potential strategy to overcome obesity and fatty liver disease. For the first time, we found a stable and long-lasting way to activate *Ces2h* through nanoparticle treatment, resulting in the regulation of lipid metabolism homeostasis. Previous research identified *Ces2c* as playing a key role in mouse lipid metabolism, while our finding showed that *Ces2h* was also involved in regulating lipid metabolism, exhibiting distinct functions that demonstrate “harmony in diversity”^45–47^. Both *Ces2c* and *Ces2h* exhibit reduced expression patterns in cases of HFD feeding, obesity, and fatty liver, similar to human *CES2*^45,47,57,59^. *Ces2h* was found to be more closely related to *CES2* than *Ces2c* by gene similarity analysis, and our study confirmed reduced *Ces2h* expression in the liver of obese mice, which was activated and restored by nanoparticles via the Nrf2/ARE-Ces2h cascade. This cascade was also applied to *CES2* but not in other *Ces2* family members. Therefore, our findings suggested that *Ces2h* was a potential homolog of human *CES2*, and that gene duplication or splitting during evolution resulted in functional divergence in the mouse Ces2 family’s lipid regulation.

In previous studies, it is a consensus that innate immunity and oxidative stress function as prime response pathways in responding to nanoparticles^50^. Although oral nanoparticle administration in our animal models mimicking lipid metabolism disease was clearly effective, a beneficial outcome of this treatment in translational medicines cannot be directly extrapolated from our results and has to be evaluated in future studies. These may also include nanoparticle-*Ces2h* signaling-based strategies designed to preferentially mediating metabolic shift in cancer, obesity, fatty liver, or hypercholesterolemia. In this regard, our study not only identified *Ces2h* as a critical target but also suggested a strategy that corrected for disorders in lipid metabolism with minimal manipulation of oxidative stress or inflammation.

Our study revealed an unusual enterohepatic antioxidant response responsible for detoxifying low concentrations of ROS at the cost of elevated levels of hepatic lipolysis. This antioxidant response can be regulated by exogenous inorganic nanoparticles via a *Ces2h*-mediated nanoparticle-ROS-Nrf2/ARE-*Ces2h* cascaded signaling pathway. The experimental work indicated that the *Ces2h* pathway played an essential role in keeping hepatic ROS homeostasis against disturbances induced by very low-dose uptake of nanoparticles in small animal models, including WT, *db/db* obesity, and HFD-fed mice. Given the ability of promoting lipid hydrolysis and subsequently restoring liver function, this process can be implemented for preventing and treating non-alcoholic fatty liver diseases of farm animals, such as cows, pigs, etc. Moreover, once the long-term safety issues of inorganic nanoparticles are addressed, our strategy can be applied in fatty liver induced metabolic disorders, such as type 2 diabetes mellitus, hypertension, and cardiovascular disease, etc^60^. In addition, due to the high expression of *Ces2h* in both liver and intestinal regions, this strategy may also be applied in some intestinal disorders induced by lipid metabolic dysfunctions^61^. It is worth noting that this study did not include experiments on a model with matched body weight loss or lean non-alcoholic steatohepatitis, which may be regarded as a limitation since weight loss may also influence metabolic parameters. Our finding provides new insights for exploring the safe uses of inorganic nanoparticles in advanced biological applications. It also provides a new direction for designing cooperative bio-intervention modulator to regulate metabolism in living systems.

## Methods

### Materials

The TiO_2_ nanoparticles were obtained from Zhonghang Nanometer Technology (Hefei, China). Cy5.5 was obtained from Amersham Biosciences (NJ, USA). Au nanoparticles and Cy5.5-modified Au nanoparticles were obtained from Biotyscience (Beijing, China). Y(CH_3_COO)_3_·xH_2_O (99.9%), sodium oleate solid (98%), 1-octadecene (90%), oleic acid (90%), fluorescent DCFH-DA probe, PD-10 columns, p‐nitrophenyl butyrate (PNPB), DF-12 complete medium, 4’,6-diamidino-2-phenylindole (DAPI), heparin sodium, 1-Naphthyl acetate, dimethylbenzene, propylene oxide, tyloxapol, hematoxylin staining solution, Fast Blue B solution, chloral hydrate, ethanol, isopropanol, glutaraldehyde, Tween‐20, H_2_O_2_, osmium tetroxide, FeSO_4_, ethylene diamine tetraacetic acid (EDTA), chloroform/methanol, [^2^H]palmitic acid, and [^2^H]cholesterol were purchased from Sigma-Aldrich. NH_4_F (99.99%), and paraformaldehyde solutions (4%) were purchased from Macklin. Malondialdehyde (MDA) assay kit were obtained from Nanjing Jiancheng Bioengineering Institute. TRIzol Reagent, 3′-(p-hydroxyphenyl) fluorescein (HPF), and phosphate buffered saline (PBS) were purchased from Invitrogen. RIPA buffer, Kanamycin, Ca^2+^- and Mg^2+^‐free Hank’s balanced salt solution (HBSS), and protease/phosphatase inhibitors were purchased from Thermo Scientific. Triglyceride quantification kit was purchased from BioVision. Dulbecco’s modified Eagle’s medium (DMEM), and 10% fetal bovine serum were purchased from Hyclone. SYBR Green Supermix, and DNase I were purchased from Roche. Random primers, and RNase H were purchased from New England Biolabs. Ni-NTA agarose, and magnetic beads containing Oligo (dT) were purchased from Qiagen. Chromatin immunoprecipitation (ChIP) assay kits, and 10% SDS-PAGE were purchased from Beyotime. CHO cells were obtained from Puhebio (Jiangsu, China). Human hepatocyte cell line LO2 was obtained from Cell Bank of Chinese Academy of Sciences (Shanghai, China). The NCTC1469 cell line was obtained from Tongpai Shanghai Biological Technology (Shanghai, China). Eukaryotic expression vectors (pcDNA3.1(+)-His-*CES2*, pcDNA3.1(+)-His-*CES2*^*g.695G>C*^, pcDNA3.1(+)-His-*Ces2h*, pcDNA3.1(+)-His-*Ces2h*^*g.436G>C*^) were obtained from General biosystem (Anhui, China). Copper grids were purchased from Zhongjingkeyi Technology (Beijing, China). The 3,3’Diaminobenzidine (DAB) solution, and Oil red O staining kit were purchased from Abcam. Collagenase IV was purchased from Zeye Biotechnology (Shanghai, China). Total ROS detection kit was purchased from Enzo Life Sciences. All the chemicals were used as received without further purification.

### Synthesis of NaYF_4_ nanoparticles

NaYF_4_ nanoparticles were synthesized by a well-established co-precipitation method^62^. In a typical experiment, Y(CH_3_COO)_3_·xH_2_O (0.4 mmol), oleic acid (5 mL), and 1-octadecene (5 mL) were mixed in a 50 mL round-bottom flask and heated to 150 °C for 60 min to form lanthanide-oleate precursor. After the solution was cooled to room temperature, sodium oleate (3.2 mmol) was added into the solution. The mixture was then heated to 100 °C for 1 h under vacuum then purged by N_2_. Thereafter, ammonium fluoride (4.4 mmol) was added followed by quickly heating the mixture to 160 °C and keeping for 1 h. The reactant was then heated to 320 °C, kept for 30 min, and cooled to room temperature. The nanoparticles were precipitated by addition of ethanol, separated by centrifugation, and washed three times with a mixture of cyclohexane and absolute ethanol. The as-prepared nanoparticles were redispersed in 4 mL cyclohexane.

### Preparation of ligand-free NaYF_4_ nanoparticles

The oleic acid coated NaYF_4_ nanoparticles in cyclohexane (1 mL) were mixed with acetone (2 mL) and precipitated by centrifugation. The precipitates were redispersed in acetone (5 mL), followed by the addition of 0.3 mL of hydrochloric acid (2 M) ultrasonication for 30 min. The nanoparticles were collected by centrifugation at 12000 × *g* for 5 min, washed with acetone several times, and redispersed in deionized water (1 mL) for further use.

### Preparation of Cy5.5-conjugated TiO_2_ nanoparticles

Cy5.5-conjugated TiO_2_ was obtained by reaction of N-hydroxysulfosuccinimide-activated TiO_2_ with amine-functionalized Cy5.5 and purification by dialysis and freeze-dry under the dark conditions^63^.

### Intrinsic esterase activity validation of inorganic nanoparticles

To investigate the intrinsic esterase activity of nanoparticles, 300 μM of 1-Naphthyl acetate was added to a 1 mL solution containing 50 μL of nanoparticles (TiO_2_, NaYF_4_, or Au at 10 mg/mL) or 1:3 diluted liver lysate, 500 μL of 0.03% Fast Blue B solution, and 450 μL of deionized water. The blank aliquot was set as deionized water with the chromogenic reagent. The absorption spectra of all the aliquots were recorded in the range of 250–500 nm using a microplate reader under 37°C conditions (Tecan Spark).

### Animal experiments

Wild-type mice, *db/db* mice, *Nrf2*-deficient (*Nrf2*^*–/–*^) mice, and *Ces2h*-deficient (*Ces2h*^*–/–*^) mice were used for animal experiments. Wild-type mice, and *db/db* mice were obtained from Model Animal Research Center of Nanjing University. *Nrf2*^*-/-*^ mice, and *Ces2h*^*–/–*^ mice were produced using the CRISPR‐CAS9 system. All mice had a C57BL/6 genetic background. Normal diet treatment was performed using normal rodent chow AIN-93M (Research Diet, New Brunswick, NJ). High fat diet (HFD) treatment was performed using a diet with 60% fat calories (Research Diet, New Brunswick, NJ). The administration of nanoparticles was performed orally by gavage. The treated doses were chosen as wide but safe dosages as low (20 μg/mouse/day), middle (50 μg/mouse/day), and high (500 μg/mouse/day) doses^64–66^. The control group was gavaged with the same volume of saline, except for flow cytometry experiment, where the vehicle solution was chosen as an equivalent volume of supernatant retrieved from fluorescence-conjugated nanoparticles centrifuged twice at 60,0000 × *g* for 60 min. Unless otherwise stated, male mice with 8 weeks were used and all mice were fasted for 5–6 h before euthanization in experiments of metabolism. Unless otherwise stated, the mice were maintained in an animal facility with a 12-h light-dark cycle, controlled temperature of approximately 22 °C, and humidity maintained at 40–60%. All animal experiments were approved by the Animal Use and Care Committee of Zhejiang University (Hangzhou, China).

### Isolation of primary tissue cells

Before isolation of hepatocytes, mice were injected with 0.5 mL heparin sodium to prevent blood clotting, anesthetized by injecting 0.2 mL 15% chloral hydrate, and performed with laparotomy. The portal vein and inferior vena cava were identified. The intravenous needle was inserted into the portal vein at a distal position, while needles and blood vessels were clamped with tweezers to prevent them from falling off. After gently releasing the needle, perfusion was then initiated for infusing 37 °C preheated HBSS. Once the liver was fully perfused, the inferior vena cava was cut to discharge the blood and perfusion fluid. The inferior vena cava was then clamped to stop the discharge when the liver lobes began to droop. After liver lobes were slowly filled up, the discharge was then performed again. These processes were repeated until 50–60 mL of solution was perfused. The liver lobes gradually swelled into a pale yellow color, and the appearance of the liver lobe standing up became inconspicuous. The solution was then changed to 5 mL 0.02% collagenase IV. The liver was then removed for liver cell isolation.

The perfused liver was transferred to a sterile glass dish filled with the basal medium, followed by the addition of DF-12 complete medium. The released cells were filtered and collected as total liver cells. Hepatocyte-enriched populations were obtained by subjecting total liver cells to two rounds of centrifugation at 50 g for 3 min at 4 °C, and collecting the resulting pellet. The supernatant from the last centrifugation was further centrifuged at 650 g (7 min, 4 °C) to collect non-parenchymal cell-enrich populations. These populations were resuspended in 10 mL HBSS, layered onto a 50%/25% two-step Percoll gradient in a 50 mL tube, and centrifuged at 1800 × *g* (15 min, 4 °C) with brake off. The mixture in the tube was divided into four parts, including debris, endothelial cell/lymphocyte-enriched populations, Kupffer cell-enriched populations, and hepatocyte/erythrocyte mixtures from top to bottom. Cell suspensions of “total liver cells”, “hepatocyte-enriched populations”, “endothelial cell/lymphocyte-enriched populations”, and “Kupffer cell-enriched populations” were selected for staining and subsequent flow cytometry.

Isolation of other tissue cells, including heart, spleen, lung, kidney, intestine, and brain, was according to a published method^67^.

### Flow cytometry

The viability of the isolated cells was checked using 4’,6-diamidino-2-phenylindole (DAPI) and incubated with fluorescent-labeled antibodies, which are cell surface antibodies including anti-CD3-PE, anti-CD19-APC, anti-CD31-Alexa Fluor 488, anti-CD68-Brilliant Violet 785 (Table S1). After being stained under dark conditions at 4 °C for 30 min, cells were washed twice in phosphate buffer saline (PBS), fixed and permeated in BD Cytofix/Cytoperm (BD Biosciences), and analyzed by a Flow Cytometer (FACSVerse; Becton Dickinson, Franklin Lakes, NJ, USA).

The gating strategies were presented in the supplementary materials (Fig. S7). Respective isotype controls were used to standardize the background fluorescence (Table S1). The singlets were chosen for cells with similar area and height in forward scatter (FSC-A vs FSC-H). Nanoparticle-positive cells were measured by their fluorescence (Cy5.5, excitation/emission: 675/694 nm) channel, with negative control set as nanoparticle-untreated, fully surface stained samples. Data were analyzed by FlowJo software (VX, Tree Star). The percentage of nanoparticle-positive cells was defined as the percentage of interested cells that fall within the nanoparticle-positive gate. Mean fluorescence intensity (MFI) represented the amount of nanoparticles absorbed by each cell. Relative MFI was obtained by normalizing MFI to the control animals to account for inter-animal variability.

### Cellular ROS determination

Cellular ROS production was determined by a fluorescent DCFH-DA probe. A reaction volume of 250 μL was set up, consisting of 10 μL freshly isolated cells from mice with/without oral nanoparticle treatment at a density of 1 × 10^4^, DCFH-DA (final concentration 20 μM), and PBS. After 30 min incubation under dark conditions, fluorescence was detected at 485 nm (excitation) and 527 nm (emission) wavelengths using a microplate reader (Tecan Spark). Negative controls consisting of untreated cells were included at each interval.

### Quantification of nanoparticles in tissues

To determine the long-term biodistribution of nanoparticles, main tissues including heart, liver, spleen, lung, kidney, intestine, and brain were collected from mice in each group after a 21-day administration period. To determine the elimination half-life of nanoparticles, tissues were excised at 0, 1, 3, 7, 14, and 21 days post-administration. The collection of lymph and blood from enterohepatic system was according to a previous work^68^. The amounts of nanoparticle elements (Ti, Au, Y) were quantified by inductively coupled plasma mass spectrometry (ICP-MS)^69^.

### Histopathological examination

Histopathological examination was performed by hematoxylin-eosin (HE) staining. Tissues were collected, fixed in 4% paraformaldehyde solutions for 4 h, and embedded in paraffin blocks. After sectioned to 4 μm, the samples were deparaffinized, and hydrated by immersing twice in dimethylbenzene (20 minutes/time), twice in 100% ethanol (5 minutes/time), and then in 75% ethanol for 5 min. The slices were then stained with hematoxylin staining solution for 5 min after water rinse. The slices were then differentiated with differentiation solutions and stained with bluing fluid. After dehydration using a series of ethanol, the slices were stained by eosin for 5 min and dehydrated with 100% ethanol thrice (5 minutes/time) and transplanted by dimethylbenzene twice (20 minutes/time), and then mounted on neutral balsam. The slices were observed using an Olympus IX71 microscope.

### Immunohistochemical examination

Paraffin-embedded liver sections with 4 μm thick were deparaffinized with xylene, rehydrated using a graded series of ethanol, and rinsed twice with PBS. Inactivation of endogenous peroxide in the liver sample was performed by 3% H_2_O_2_. Sections were heated at 95 °C in citric acid antigen retrieval buffer for 20 min, and then cooled at room temperature for 20 min. Primary antibody (Nrf2, Cell Signaling Technology, Cat#12721, 1:200) was added to sections and incubated at 37 °C for 70 min. After rinsed with 0.2 M PBS, sections were incubated with HRP conjugated secondary antibody for 30 min at 37 °C. Sections were rinsed, incubated with DAB solution for 3 min at room temperature, and were counterstained with hematoxylin. Sections were observed with an Olympus IX71 microscope. The staining intensity for each sample was quantified by average optical density using ImageJ software.

### Oil red O staining

Liver samples were collected and fixed in 4% paraformaldehyde overnight at 4 °C. To detect neutral lipids, oil red O staining was performed. Briefly, samples were washed with PBS and incubated with 0.3% oil red O solution in 60% isopropanol for 1 h. The samples were rinsed with 60% isopropanol and washed with PBS containing 0.1% Tween‐20. Sections were observed using an Olympus IX71 microscope.

### Entry of nanoparticles into the tissue

Tissues were collected, and fixed with 2.5% glutaraldehyde overnight. After further fixed with 1% osmium tetroxide for 2 h, tissues were dehydrated with a series of graded ethanol solutions (30%, 50%, 70%, 80%, 90%, and 95%; 15 minutes/step) and then dehydrated with 100% ethanol for 20 min. The tissues were infiltrated with propylene oxide, embedded in the resin and sliced into ultrathin sections (75 nm). The sections were mounted on copper grids and observed using a transmission electron microscope (TEM, Hitachi Model H-7650, Hitachi, Japan) to analyze the entry of nanoparticles into the tissue.

### Hepatic RNA sequencing

After extracting total RNA from the liver (which was quality assessed using the Agilent 2100 Bioanalyzer, with a RNA integrity number >8.5) and digesting DNA with DNase I, eukaryotic mRNA with a poly-A tail was enriched using magnetic beads containing Oligo (dT). The bound mRNA was eluted and denatured using bivalent cations and high-temperature conditions. The reverse transcription process was performed using random primers to yield first-strand cDNA. The second strand of cDNA was synthesized by digesting the mRNA strand in the heterozygous double strand by RNase H. After the purification of two-strand cDNA using magnetic beads, ends were repaired, a 3’end A was added, the joint was connected, and then the fragment size was selected. Finally, PCR amplification was performed. An Illumina Hi-Seq X ten platform was used for sequencing. After RNA sequencing data processing, genome alignment, and function annotation, differential expression analysis (based on Transcripts Per Million) and functional enrichment analysis were performed using edgeR and enrichR, respectively^70,71^. A gene was considered differentially expressed if *P* < 0.05 and fold change >2 or < −2. The sequencing raw data has been deposited in NCBI with accession number PRJNA640462.

### Gene expression analysis

RNA was isolated from tissue samples by TRIzol Reagent. Expressions of mRNA (Table S2) were analyzed by quantitative reverse‐transcription polymerase chain reaction (RT‐qPCR) on a 7500 real‐time PCR machine from Applied Biosystems (Foster City, CA) using SYBR Green Supermix. Results were calculated using cycle threshold values and normalized to *Gapdh* mRNA level.

### ChIP assays

ChIP assays were performed on liver samples using ChIP assay kits. Chromatin lysate was precleared and immunoprecipitated with a specific antibody (Nrf2, Cell Signaling Technology; Cat#12721, 1:200) or immunoglobulin G (Abcam, Cat#ab6709, 1:200). After washing and elution, immunoprecipitates were heated at 65 °C overnight to reverse cross‐linking of DNA and protein. Then, qPCR was performed to amplify protein-bound DNA. The chromatin region-specific primers used for ChIP qPCR was presented in the Table S3. The positive control was set as ARE of the NADP(H) quinone oxidoreductase 1 (Nqo1) promoter, which is a known target of Nrf2. The negative control was set as a non-specific region located 2 kilobases away from this ARE.

### Western blotting analysis

Total proteins were extracted from frozen tissue by homogenization using RIPA buffer containing protease/phosphatase inhibitors. The primary antibodies used for western blotting analysis were Nrf2 (Cell Signaling Technology, Cat#12721, 1:1000) and Ces2 (Abcam, Cat#ab215042, 1/1000). The bands were visualized by ECL Advance Western Blotting Detection Reagents (GE Healthcare, Little Chalfont, UK) and quantified by ImageJ (National Institutes of Health, Bethesda, MD).

#### Molecular docking between cholesterol ester, ROS, and carboxylesterase

##### Homology modeling of human CES2 protein and mouse Ces2h protein crystal structure

We modeled crystal structures of human CES2 and mouse Ces2h using Modeller (v9.18) based on CES1, which is a homologous protein of known crystal structure. The primary protein sequences of CES2 and Ces2h were obtained by the public protein data bank (UniProt) and listed as followed:

> HUMAN Carboxylic ester hydrolase CES2

PLTPCPVQTPRLGKALIHCWTDPGQPLGEQQRVRRQRTETSEPTMRLHRLRARLSAVACG

LLLLLVRGQGQDSASPIRTTHTGQVLGSLVHVKGANAGVQTFLGIPFAKPPLGPLRFAPP

EPPESWSGVRDGTTHPAMCLQDLTAVESEFLSQFNMTFPSDSMSEDCLYLSIYTPAHSHE

GSNLPVMVWIHGGALVFGMASLYDGSMLAALENVVVVIIQYRLGVLGFFSTGDKHATGNW

GYLDQVAALRWVQQNIAHFGGNPDRVTIFGESAGGTSVSSLVVSPISQGLFHGAIMESGV

ALLPGLIASSADVISTVVANLSACDQVDSEALVGCLRGKSKEEILAINKPFKMIPGVVDG

VFLPRHPQELLASADFQPVPSIVGVNNNEFGWLIPKVMRIYDTQKEMDREASQAALQKML

TLLMLPPTFGDLLREEYIGDNGDPQTLQAQFQEMMADSMFVIPALQVAHFQCSRAPVYFY

EFQHQPSWLKNIRPPHMKADHVKFTEEEEQLSRKMMKYWANFARNGNPNGEGLPHWPLFD

QEEQYLQLNLQPAVGRALKAHRLQFWKKALPQKIQELEEPEERHTEL

> MOUSE Carboxylic ester hydrolase Ces2h

MRLEQIHARLTTATCGLLLLLRVQGQDSTSPIRTTHTGQILGSLIHMKDLDVGVHSFLGI

PFAKPPVGPLRFAPPEPPEPWGGVRDGTSHPAMCLQDITAMNMQAFKLLKLTLPPFPMSE

DCLYLNIYAPDHAHEGSNLPVMVWIHGGSLVIGMASMYDGSMLAAMENVVVVTIQYRLGV

LGFFSTGDERARGNWGYLDQVAALRWLQQNIAYFGGNPDRVTIFGTSAGGTSVSSLVVSP

MSQGLFRGAIMESGVALISSLISVSSDVVYQTVANLSGCEQVDSEALVNCLRGKSEEEIM

AINKAFKIIPGIVDGIFLPRHPQELMASADFHPVPSIIGVNNDEYGWIIPSSMSMIDSKK

GMDRQMVQAILQRRATQMMWPPEVSDLLMEEYMGDNEDPQFLQVQFKEMMKDFTFVIPAL

QVAQFQRSHAPVFFYEFQHRPSFFKDSKPSHVKADHGDEILFIFRSFWGGTQVDFTEEEE

LLSRRMMKYWANFARQRNPNSEGLPYWPMFDQDEQYLQLDTQPAVGRALKTRRLQFWTKT

LPEKIQELKDIEDRHKEL

The crystal structure of CES1 was obtained from the Protein Data Bank (PDB ID: 3K9B). Modeling criteria were set as follows: 1. the sequence similarity of protein structures was more than 30%; 2. the structures had similarity characteristics. We additionally modeled point-mutated protein structures of CES2 (HGG^193^A substituted by HGA^193^A, CES2^p.G193A^) and Ces2h (HGG^148^S substituted by HGA^148^S, Ces2h^p.G148A^) by Pymol (v2.3). The 500 ps non-limiting molecular dynamics analysis was performed on the mutated models using Amber 16. The molecular dynamics parameters were as follows: protein is not added by heavy atom to perform a position restriction and kept in semi-flexible. The average structure was extracted based on the trajectory of molecular dynamics and was used for subsequent analysis.

##### Structure optimization

A molecular dynamics simulation analysis is required to optimize the modeling results by Amber16 to obtain reliable three-dimensional structure of proteins. In order to achieve the fittest protein structure, molecular dynamics simulation experiments need to be repeated for kinetic reliability. The structure was superimposed and supplemented, and the molecular dynamics of the conformation protein was optimized for 1.0 ns until the structure was balanced. After molecular dynamics was balanced, the average structure was saved for subsequent analysis and docking.

##### Pocket identification and pocket analysis based on similar crystal structure

The binding pocket is a ligand binding site based on biological macromolecules and it plays a decisive role in the drug development process. In protein-protein and protein-molecule interactions, docking pocket amino acids play a most intuitive guiding role in explaining the mechanisms of protein function. Thus, we identified the binding pockets of CES2 and Ces2h proteins, which consist of the serine hydrolase catalytic triad (Ser^272^, Glu^389^, and His^502^ in CES2; Ser^227^, Glu^344^, His^457^ in Ces2h) and the oxyanion hole (HG^192^G^193^A in CES2 and HG^147^G^148^S in Ces2h). In the protein crystal structure database, we found their sequence structural analog, human carboxylesterase in complex with Coenzyme A (PDB ID: 2H7C), and used sequence alignment to get an overview of the interaction pattern between CES2/Ces2h and the ligand.

##### Molecular docking

Protein-molecule docking was analyzed by AutoDock (v4.2.6) using flexible docking to obtain the preliminary docked phase structure. At least 50 docking results were generated during docking, and the phase with the best docking energy was selected for structural extraction for subsequent molecular dynamics studies. The process of AutoDock docking is as follows: 1. Use amino acid residues around the active site of the receptor to form a large Box, and then use different types of atoms as probes to scan and calculate the energy of grid points, all of which is completed by the AutoGrid program; 2. Conformational search of ligands within the Box is performed by the AutoDock program. Scoring is performed according to conformation, orientation, position, and energy of ligands, and finally the results are ranked.

##### In vitro CES2/Ces2h protein expression and purification

CES2/Ces2h and CES2^p.G193A^/Ces2h^p.G148A^ proteins were produced by transfecting CHO cells with the corresponding eukaryotic expression vectors (pcDNA3.1(+)-His-*CES2*, pcDNA3.1(+)-His-*CES2*^*g.695G>C*^, pcDNA3.1(+)-His-*Ces2h*, pcDNA3.1(+)-His-*Ces2h*^*g.436G>C*^). Eukaryotic expression vectors were transfected into CHO cells by electroporation with two electric shocks, using 160 V for 15 min in the ice bath environment, with a 1-min interval. The electro-transfected cells were added to DMEM medium, and kanamycin was added for selective pressure screening. After transfection for 48 h, cells were washed with cold PBS and lysed with protein lysate buffer containing protease inhibitors for 30 min. The soluble fractions were collected after centrifugation at 12000 rpm/minute for 20 min at 4 °C for subsequent protein quantitative detection on a 10% SDS-PAGE followed by Coomassie Blue staining. For affinity purification, the cell culture solution was centrifuged at 4750 × g for 8 min at 4 °C. The centrifuged supernatant was then filtered by plate filter after concentration using a 6 × 10^3^ hollow fiber column, and then affinity purified on Ni-NTA agarose. After elution, the imidazole was removed with PD-10 columns. The purified proteins were analyzed using Coomassie Blue staining and western blotting (primary antibody: 6*His, His-Tag Mouse Monoclonal antibody, Proteintech 66005-1-Ig; secondary antibody: HRP-conjugated 6*His, His-Tag Mouse Monoclonal antibody, Proteintech HRP-66005).

##### CES2/Ces2h catalytic kinetics influenced by nanoparticles

Human hepatocyte cell line LO2 or NCTC1469 cell line derived from normal mouse liver were cultured in DMEM with 10% fetal bovine serum, 1% antibiotics and grown in a 5% CO_2_ atmosphere at 37 °C. Endogenous expressed CES2/Ces2h was knockout using the CRISPR‐CAS9 system. Then, mutated CES2^p.G193A^/Ces2h^p.G148A^ LO2/NCTC1469 cell lines were produced by transfecting LO2/NCTC1469 cells with the corresponding eukaryotic expression vectors (pcDNA3.1(+)-His-*CES2*^*g.695G>C*^, pcDNA3.1(+)-His-*Ces2h*^*g.436G>C*^). For static lipid analysis, cells were incubated for the indicated duration in medium containing 800 μM palmitic acid (Sigma-Aldrich, O3008) for 6 h. After 48 h of treatment with either PBS or nanoparticles, the cells were harvested for lipid extraction. The cellular levels of triglyceride (Wako), free fatty acid (NEFA C Test Kit, Wako), free cholesterol and cholesterol ester (Amplex Red Cholesterol Assay Kit, Invitrogen), and phospholipid (Wako), were measured using commercial kits, following the manufacturer’s instructions. For lipid turnover analysis, a pulse-chase experiment design was applied. Cells were pulsed with 800 µM [1-^13^C]oleate bound to fatty acid-free bovine serum albumin in a 3:1 molar ratio for 1.5 h at 48 h postplating for PBS or nanoparticle treatments. Following the pulse, lipid extractions were obtained from a portion of cells to determine the level of labeled fatty acid incorporation into cellular lipid fractions, as described below. The remaining cells were washed with PBS and then cultured in fresh medium without labeled oleate for an additional chase period. After the chase period, the cells were collected and lipids were extracted. Analysis of cellular lipid fractions incorporating labeled fatty acids was performed on the Agilent 6490 triple quadrupole liquid chromatography-mass spectrometry instrument using reverse-phase chromatographic separation and mass spectrometry with a targeted dynamic multiple reaction monitoring method^72^. Cellular lipid fractions were quantified by integration, followed by normalization against internal standards^72^. The molar amount of ^13^C-lipids by class is the sum of the molar amounts for all quantified individual ^13^C-lipids in class. The production of intracellular ·OH was determined by fluorescent reporter dye HPF (Ex/Em=492/515 nm) at a concentration of 5 μM.

For CES2/Ces2h enzyme catalytic analysis, CES2/Ces2h and mutated CES2^p.G193A^/Ces2h^p.G148A^ proteins were produced by transfecting CHO cells with the corresponding eukaryotic expression vectors. Expressed proteins were affinity-purified by Ni Sepharose Fast Flow, and analyzed using 10% SDS-PAGE followed by Coomassie Blue staining. The enzymatic activities of CES2/Ces2h and mutated CES2/Ces2h were measured using PNPB according to a previous study^59^. As enzyme catalysis system cannot simulate the intracellular ·OH generation, a Fenton reaction was applied to generate ·OH in enzyme catalysis system, consisted of FeSO_4_ (50 μM), EDTA (30 μM), PBS (pH 7.2, 4 mM), and H_2_O_2_ (500 μM)^73^. To detect ·OH formation, we used the HPF at a concentration of 5 μM.

##### Fatty acid oxidation

For in vivo experiments, fatty acid oxidation was assessed by [^2^H]palmitic acid and [^2^H]cholesterol injection and measuring the production of ^2^H-labeled water. For in vitro experiments, fatty acid oxidation was measured by the production of β-hydroxybutyrate using the commercial kit (Cayman) according to the manufacturer’s instructions.

##### Tissue lipid analysis

Liver samples were homogenized in methanol to further extract lipids in chloroform/methanol (2:1 v/v) according to a previous study^74^. The concentrations of hepatic TG and cholesterol were determined by Infinity reagents from Thermo Scientific (Waltham, MA). For in vitro analysis, hepatocytes were harvested from mice with/without oral nanoparticle treatment and measured for TG concentrations by a triglyceride quantification kit.

##### Tissue MDA analysis

The MDA content in tissues were examined by a MDA assay kit. Proteins from the same sample were used as standardized internal references for tissue volume.

##### VLDL secretion

The effect of nanoparticles on the VLDL secretion was performed as a previous work^75^. Briefly, wild-type C57BL/6 mice with or without nanoparticle treatment were fasted overnight and injected intravenously with Tyloxapol (500 mg/kg), an inhibitor for lipoprotein lipase. The blood was taken at 0, 30, 60, 90 min after injections, and determined for plasma TG concentrations.

##### Energy metabolism monitoring

The comprehensive lab animal monitoring system (Columbus Instruments) was used to determine 24‐h O_2_ consumption, CO_2_ production, respiration quotient rate (RER), and food intake.

### Statistics and reproducibility

Comparisons among three or more groups were performed by one-way analysis of variance and the L.S.D. test using SAS (v9.21, Cary, USA). Comparisons between two groups were performed by two-sided student’s *t* test using SAS. Significance was defined as *P* < 0.05. All data were presented as mean ± SEM. Results from representative experiments (such as micrographs) are repeated at least 3 times independently with similar results.

### Reporting summary

Further information on research design is available in the Nature Portfolio Reporting Summary linked to this article.

## Supplementary information

Supplementary Information

Reporting Summary

## Data Availability

The sequencing raw data generated in this study have been deposited in the NCBI database under accession code PRJNA640462. All relevant data that support the findings of this work are available in the Supplementary Information or Source Data file. Source data are provided with this paper.

## Source data

Source Data
