# Supplementary material for: Antioxidant hepatic lipid metabolism can be promoted by orally administered inorganic nanoparticles
Source: Nat Commun. 2023 Jun 20;14:3643. doi: 10.1038/s41467-023-39423-3 (PMC10281969; doi:10.1038/s41467-023-39423-3)
Supplement: Supplementary file 1 — Supplementary Information [file 41467_2023_39423_MOESM1_ESM.pdf]

Supplementary Information

Antioxidant hepatic lipid metabolism can be promoted by orally administered inorganic nanoparticles

Jie Cai[1,2†*], Jie Peng[3†], Juan Feng[1†], Ruocheng Li[1], Peng Ren[1], Xinwei Zang[1], Zezong Wu[1], Yi Lu[1], Lin Luo[1], Zhenzhen Hu[1], Jiaying Wang[4], Xiaomeng Dai[5], Peng Zhao[5], Juan Wang[4], Mi Yan[3], Jianxin Liu[1], Renren Deng[3,5*], Diming Wang[1*]

[1]*College of Animal Sciences, Dairy Science Institute, MOE Key Laboratory of Molecular Animal Nutrition, Zhejiang University, Hangzhou 310029, PR China*

[2]*Department of Veterinary Medicine, College of Animal Sciences, Zhejiang University, Hangzhou, 310029, PR China*

[3]*State Key Laboratory of Silicon and Advanced Semiconductor Materials, Institute for Composites Science Innovation, School of Materials Science and Engineering, Zhejiang University, Hangzhou 310027, China*

[4]*Institute of Environmental Health, MOE Key Laboratory of Environmental Remediation and Ecosystem Health, College of Environmental & Resource Sciences, Zhejiang University, Hangzhou 310058, PR China*

[5]*Department of Medical Oncology, The First Affiliated Hospital, School of Medicine, Zhejiang University, Hangzhou 310003, PR China*

[†]These authors contributed equally to this work.

*E-mail: zjcaijie@zju.edu.cn; rdeng@zju.edu.cn; wdm@zju.edu.cn

Supplementary Table S1. Antibodies used for cytometry.

Antibody	Company	Category number	Applied dose
PE Rat Anti-Mouse CD3	Abcam	Ab22268	1:100
APC Rat Anti-Mouse CD19	Abcam	Ab25484	1:100
Alexa Fluor 488 Rat Anti-Mouse CD31	Bio-Rad	MCA2388A488T	1:100
Brilliant Violet 785 Rat Anti-Mouse CD68	BioLegend	137035	1:100
DAPI	Becton Dickinson	564907	1:20000
PE Rat IgG2a, Isotype Control	Abcam	Ab253047	1:100
APC Rat IgG2a, Isotype Control	Abcam	Ab239461	1:100
Alexa Fluor 488 Rat IgG2a, Isotype control	Bio-Rad	MCA1212A488	1:100
Brilliant Violet 785 Rat IgG2a, κ Isotype control	BioLegend	400274	1:100

Supplementary Table S2. Primers used for RT-qPCR experiments

Name	Forward (5’ -> 3’)	Reverse (5’ -> 3’)
Apob	TCACCATTTGCCCTCAACCT	ATCCAGGAGAGGTGAAAGAACATT
Ces2a	GTGGACTGGTTGTAGGATCAGC	TTCTTCTGCACCCAGCGTAAG
Ces2b	AACGATGAGTTTGGTTGGACC	GAGGCAGCATCAGTTGTGC
Ces2c	GCCAACCCCATCAGAAACACA	TTCAGCATGTCAAGATTTTGCAG
Ces2d	CTTAGTCTTCCAAGCAGCCCA	AAAAGCACAGCATTCAGCCAG
Ces2e	CCGAGTCACCATTTTTGGAGA	GGAGATAAGATCAGGGAGCACA
Ces2f	GCCTACCATTATACCTGACTCCC	CACAGCAGGCATAAACCTGAA
Ces2g	AGGTCCAAGGCAGGCTCAT	GGCCCTCCATATTCATCGTAACA
Ces2h	GAATCTAAGCCCCACAGGCAT	GGGTTGGTTACACAGGGCTA
Mttp	TGGGTTTTGCGGGAATGGT	TGAGAGGCCAGTTGTGTGAC
Acaca	CGGACCTTTGAAGATTTTGTGAGG	GCTTTATTCTGCTGGGTGAACTCTC
Acacb	TTCATGGACAGTGGCTTCTC	GCACGCCTTACTGAAGAGAAG
Mecr	CCCAGTCCTTCACTTCTATGTTC	GTAGCACAGTTCAGTCTCGAC
Fasn	AGACCCGAACTCCAAGTTATTC	GCAGCTCCTTGTATACTTCTCC
Srebf1	GCCCCTGTAACGACCACTG	GCCCCTGTAACGACCACTG
Srebf2	TGGGCGATGAGCTGACTCT	CAAATCAGGGAACTCTCCCAC
Acot8	GGTCTGGGAGATGCTCATGG	CCCAGTAATGCCTTCCTCTGT
Acox1	TCGCAGACCCTGAAGAAATC	CCTGATTCAGCAAGGTAGGG
Cpt1b	GCACACCAGGCAGTAGCTTT	CAGGAGTTGATTCCAGACAGGTA
Ehhadh	CCGGTCAATGCCATCAGT	CTAACCGTATGGTCCAAACTAGC
Atgl	ATGTTCCCGAGGGAGACCAA	GAGGCTCCGTAGATGTGAGTG
Dgat1	GTGCCATCGTCTGCAAGATTC	GCATCACCACACACCAATTCAG
Dgat2	GCGCTACTTCCGAGACTACTT	GGGCCTTATGCCAGGAAACT
Gpat1	CTTGGCCGATGTAAACACACC	CTTCCGGCTCATAAGGCTCTC
Gpat2	AGCAGAGGAGTAACCACAATGG	GGGCGATACTTTCCCAGGA
Gapdh	GGTCATCATCTCCGCCCC	TTCTCGTGGTTCACACCCATC

Supplementary Table S3. Primers used for ChIP experiments

Name	Forward (5' -> 3')	Reverse (5' -> 3')	Reference
*Nqo1*ARE	AGCAGAACGCAGCACGAAT	CACTCAGCCGTGGGAAGT	Rushmore et al., 1991
*Nqo1*NS	TACGCTGTAGTGGTGGTGGA	TCTGGGGACTTGGGTATCTG	Ulrike et al., 2013
Ces2h	TCGATTGCAGATATTCACCTGG	TCGATTGCAGATATTCACCTGG	-

NS: non-specific

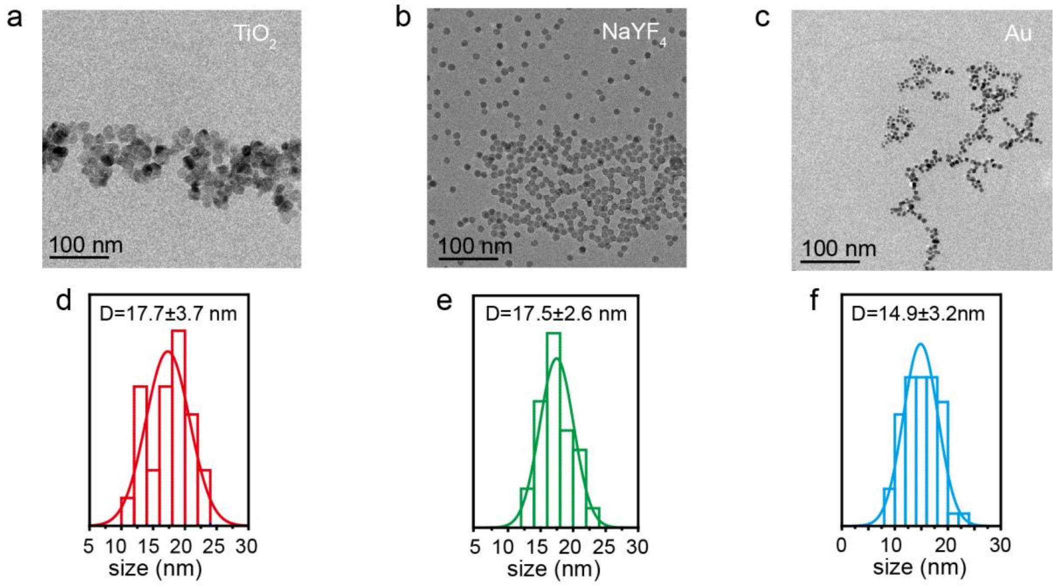

Supplementary Fig. 1 | a-c, TEM images and **d-f**, corresponding size distributions of TiO_2, $NaYF_4$, Au nanoparticles, respectively.

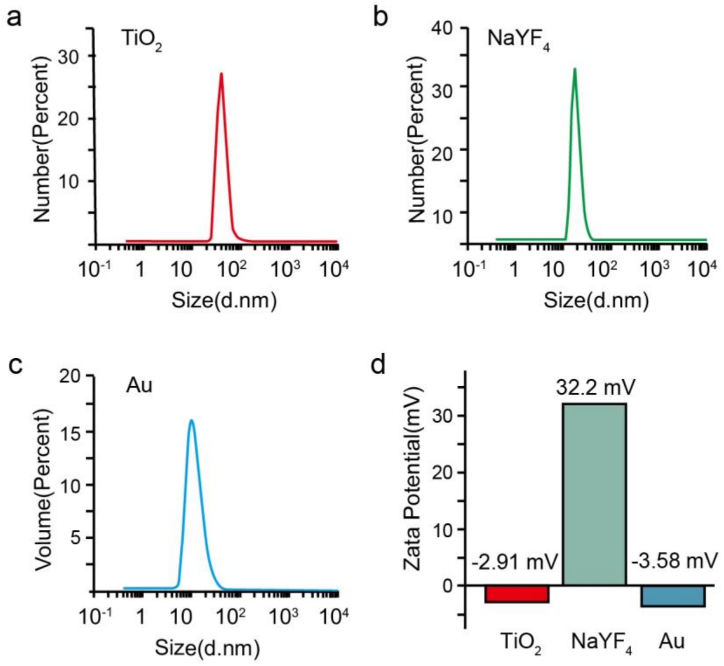

Supplementary Fig. 2 | Dynamic light scattering (DLS) measurements indicating hydrodynamic particle size distribution of (**a**) TiO_2, (**b**) $NaYF_4$, and (**c**) Au nanoparticles in aqueous solution, respectively. **d**, Corresponding zeta potentials of these nanoparticles.

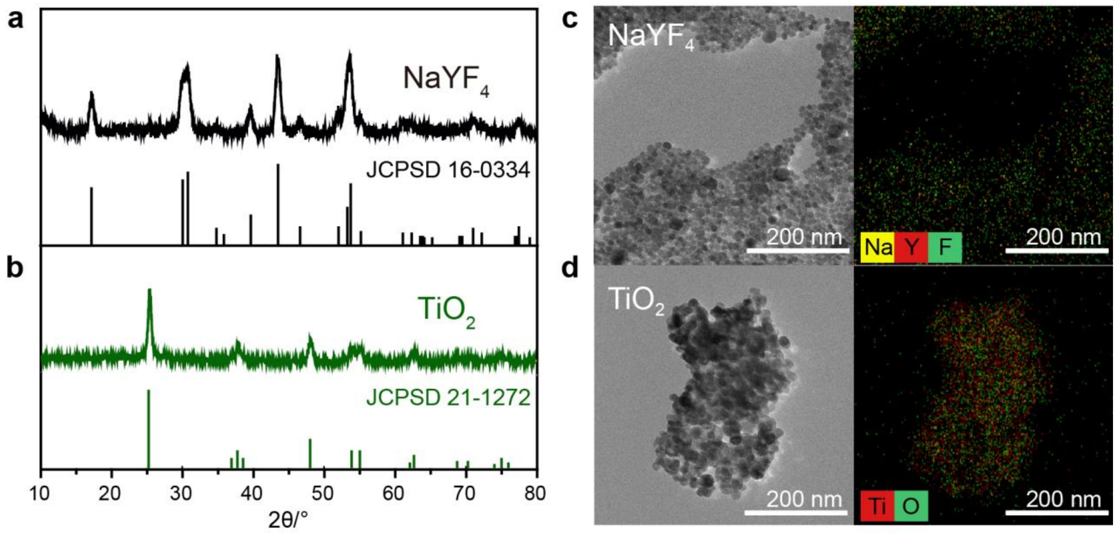

Supplementary Fig. 3 | X-ray diffraction (XRD) patterns of as-prepared (**a**) $NaYF_4$ nanoparticles and (**b**) TiO_2 nanoparticles, respectively. The XRD patterns indicate a hexagonal-phase of $NaYF_4$ and tetragonal phase of TiO_2 nanoparticles, respectively. **c**, EDS elemental mapping of $NaYF_4$ indicating the contain of Na, Y and F in the material. **d**, EDS elemental mapping of TiO_2 indicating the presence of Ti and O in the nanoparticles.

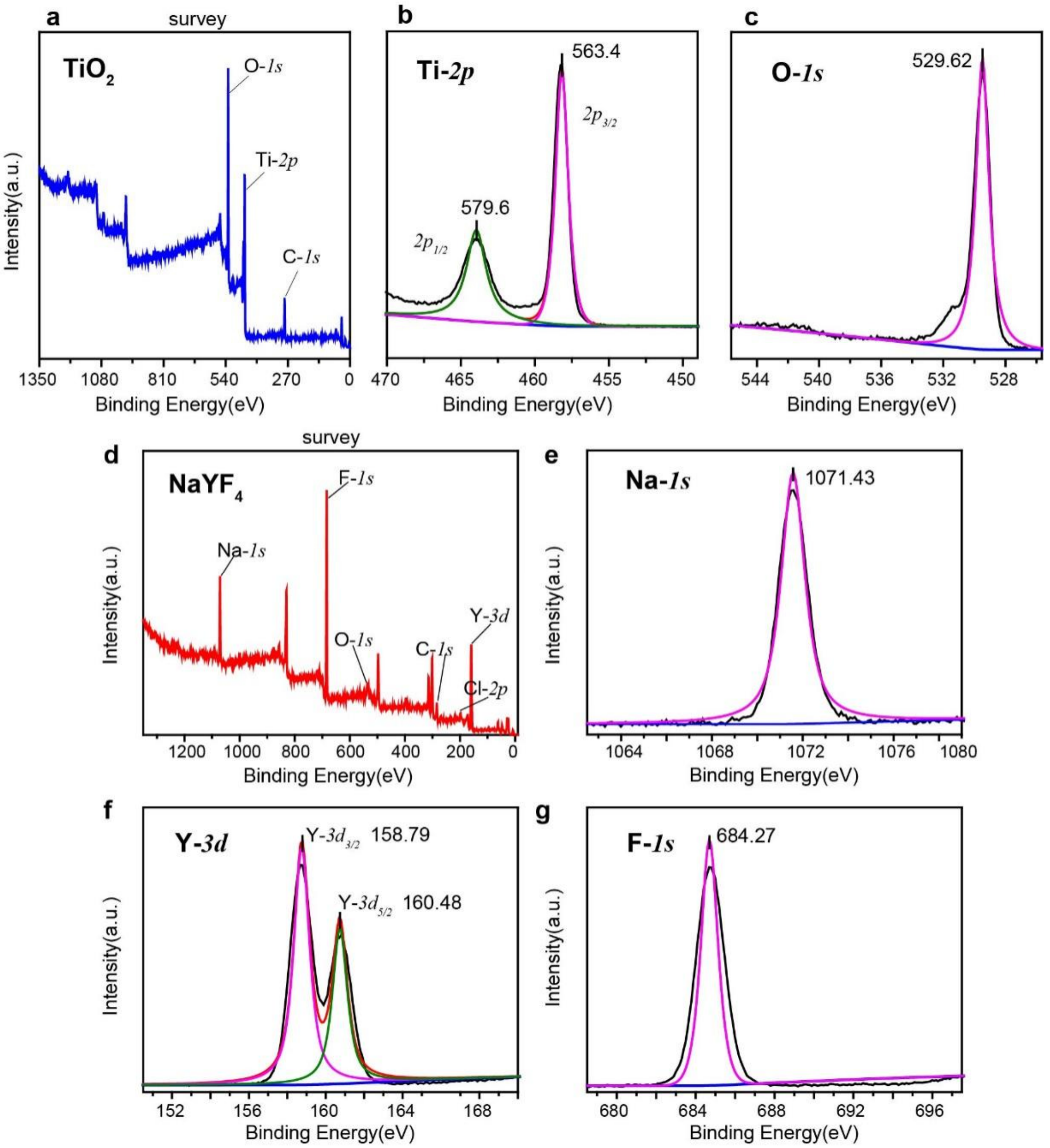

Supplementary Fig. 4 | XPS spectra of TiO_2 (**a-c**) and $NaYF_4$ (**d-f**).

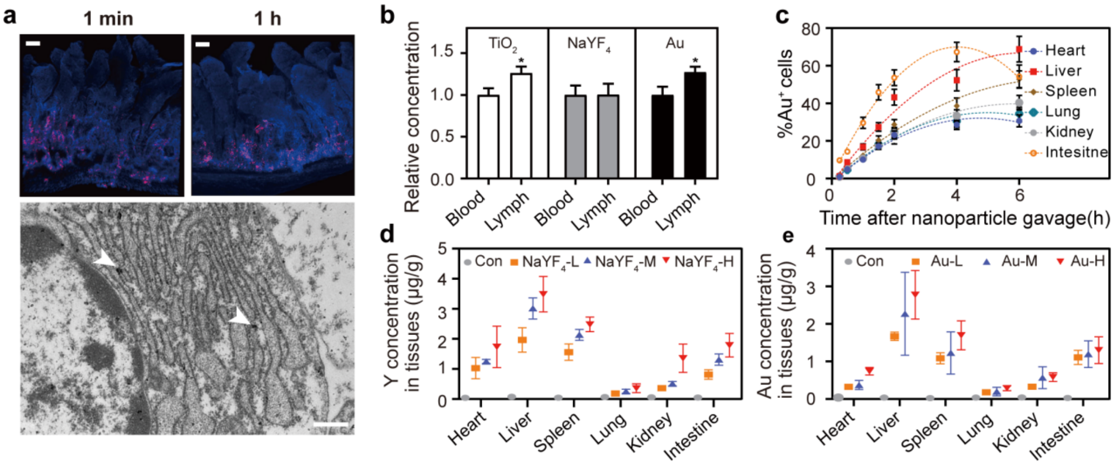

Supplementary Fig. 5 | Intestinal transmissivity, circulatory transport, and tissue enrichment of nanoparticles. a, 3D imaging of the oral TiO_2 nanoparticles penetrating the intestine at 1 minute and 1 hour after oral administration (top). Scale bar, 100 μm. The transmission electron microscopy (TEM) image (bottom) depicted the persistence of TiO_2 nanoparticles in the enterocytes after single oral administration (0.72 mg/kg). White arrows indicated the nanoparticles. Scale bar, 500 nm. **b**, Relative concentration of nanoparticles in the blood and lymph after a single oral administration (0.72 mg/kg). * represented $P < 0.05$. n = 6. **c**, Percentage of Au nanoparticle-positive cells in the main organs of mice gavaged by Cy5.5-conjugated Au nanoparticles (0.72 mg/kg). The control group was set as mice gavaged by a same volume of the vehicle solution chosen as supernatant retrieved from Cy5.5-conjugated Au nanoparticles centrifuged twice at 600000 g for 60 minutes. n = 6. The full gating strategy was included in Fig. S6. **d-e**, The bio-distributions of Y and Au in the main organs of mice were measured at high dose (18 mg/kg/day), middle dose (1.8 mg/kg/day), and low dose (0.72 mg/kg/day). n = 6. Data in **b-e** are presented as mean values ± SEM. Source data are provided as a Source Data file.

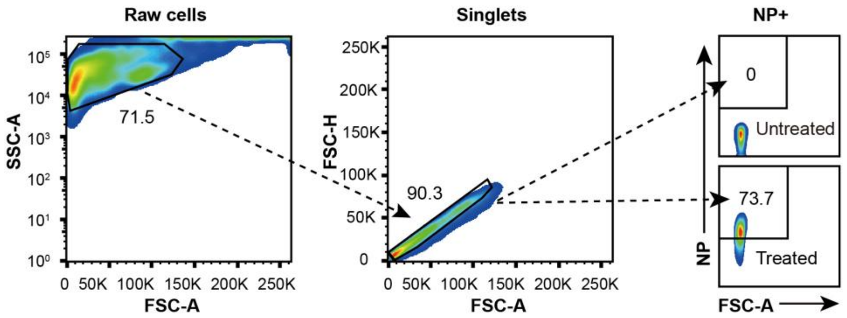

Supplementary Fig. 6 | Flow cytometry gating strategy used to identify nanoparticle-positive cells in *in vivo* experiments. Total cells from each organ were obtained from mice with/without nanoparticle treatment. Debris was removed by FSC vs SSC gating. The singlets were identified by FSC-A vs FSC-H gating. The representative plots took Cy5.5-conjugated Au nanoparticle as an example.

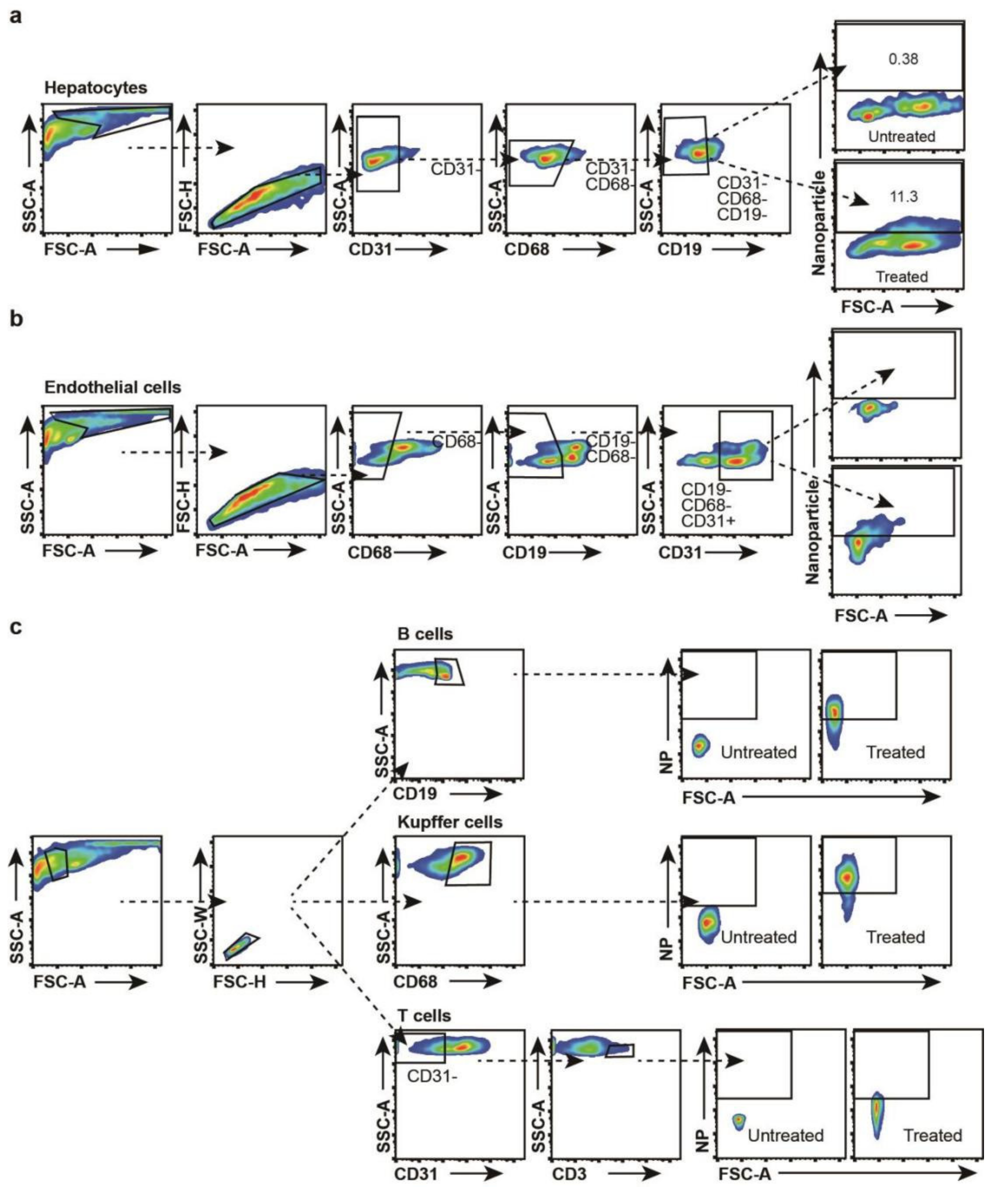

Supplementary Fig. 7 | Flow cytometry gating strategy used to identify nanoparticle-positive liver cells. Total liver cells were obtained from mice with/without nanoparticle treatment after liver perfusion, enzyme digestion, and centrifugation. Debris was removed by FSC vs SSC gating. Hepatocytes (**a**), endothelial cells (**b**), B cells, Kupffer cells, and T cells (**c**) were identified in their respective enriched subpopulations with surface markers CD31, CD68, and CD19. Gating strategy used for cell surface marker expression was based on fluorescent background of unstained liver cells. The representative plots took Cy5.5-conjugated Au nanoparticle as an example. Percentage of nanoparticle-positive cells was calculated as nanoparticle^{+} cells (%) = nanoparticle^{+} cells$_{\text{nanoparticle-treated}}$ (%) - nanoparticle^{+} cells$_{\text{nanoparticle-untreated}}$ (%). Nanoparticle uptake ability was evaluated by mean fluorescence intensity. The nanoparticle-untreated mice were gavaged by a same volume of the vehicle solution chosen as supernatant retrieved from Cy5.5-conjugated Au nanoparticles centrifuged twice at 600000 g for 60 minutes.

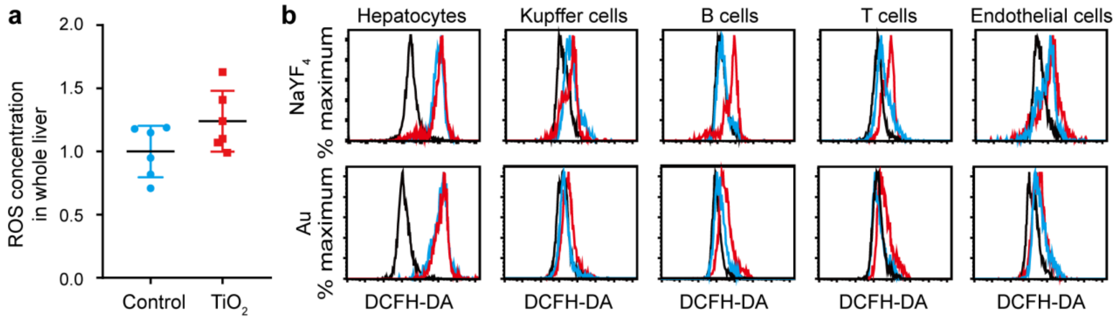

Supplementary Fig. 8 | Subcellular localization of nanoparticles during long-term retention in hepatocytes and their effect on cellular ROS. a, ROS concentration in whole liver perspective influenced by TiO_2 nanoparticle gavage (0.72 mg/kg). The liver samples (n = 6) were collected 24 hours after gavage. Total ROS in liver cells without subpopulation identification was determined with a total ROS detection kit. Data are presented as mean values ± SEM. **b**, ROS generation within different liver cell types from mice administered with $NaYF_4$ and Au nanoparticles (0.72 mg/kg). Liver samples were collected 6 (black), 18 (blue), and 24 (red) hours after nanoparticle gavage. The full gating strategy was included in Fig. S7. Source data are provided as a Source Data file.

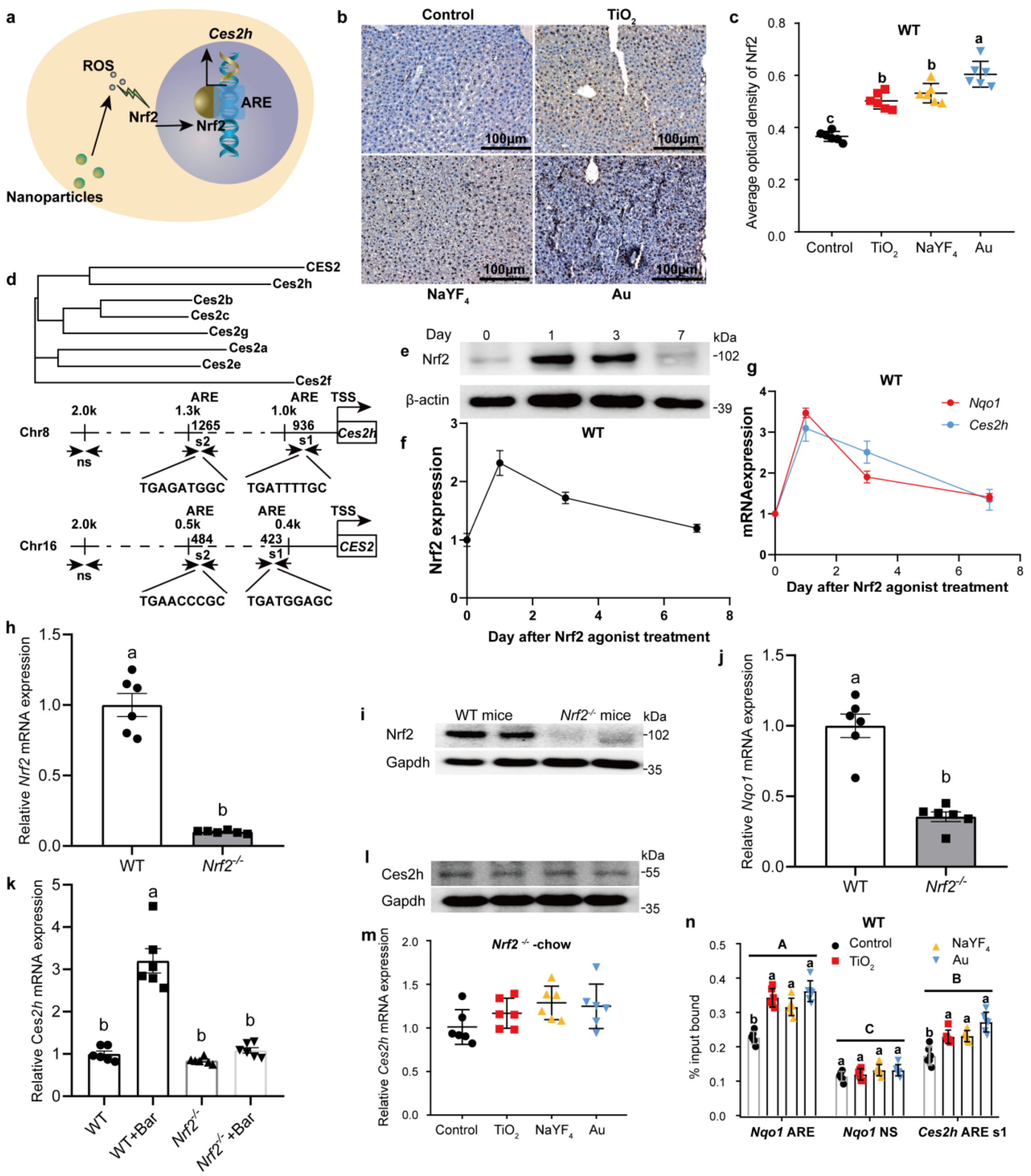

Supplementary Fig. 9 | Effects of nanoparticles on hepatic Nrf2-*Ces2h* cascade. **a**, Working hypothesis of hepatic Nrf2 response to nanoparticles in regulating *Ces2h* expression. Cellular reactive oxygen species (ROS) is generated after nanoparticle entry. Nrf2 responds to the ROS and is translocated to nucleus where it binds to the anti-oxidative response element (ARE). As a result, ARE-containing *Ces2h* gene is activated. **b-c**, Wild-type mice fed with chow food were gavaged with either saline or nanoparticles (TiO_2, $NaYF_4$, and Au; 0.72 mg/kg). After 21 days, liver samples were collected and Nrf2 expression was determined by immunohistochemistry (IHC). Representative liver images are shown in (**b**). Nrf2 expression in IHC images was analyzed by average optical density through ImageJ and shown in (**c**). **d**, Similarity comparison of mouse *Ces2* family and human *CES2* (top). Data from NCBI, where

Ces2d was not found. Region upstream of the mouse *Ces2h* (more similar to human *CES2* than other mouse *Ces2* genes) and Human *CES2* (bottom). Chr represents the chromosome; TSS represents the transcriptional start site; Arrows represent binding sites of the primers used for amplification of the two AREs (s1 and s2), or non-specific region away from the AREs. **e-g**, Protein expression of Nrf2 (e-f), and mRNA expression of its target *Nqo1* and *Ces2h* (g) in liver after Nrf2 agonist treatment (Bardoxolone, 3 mg/kg). **h-i**, mRNA (h) and protein (i) levels of Nrf2 in wild-type mice and *Nrf2*$^{-/-}$ mice. **j**, Nrf2 targeted gene—*Nqo1* mRNA levels in wild-type mice and *Nrf2*$^{-/-}$ mice. **k**, mRNA expression of *Ces2h* in liver after Nrf2 agonist treatment (Bardoxolone, 3 mg/kg) in wild-type mice and *Nrf2*$^{-/-}$ mice. **l-m**, Ces2h protein expression (l) and *Ces2h* mRNA expression (m) measured by RT-qPCR in *Nrf2*$^{-/-}$ mice fed with chow food and gavaged with either saline or nanoparticles (TiO_2, $NaYF_4$, and Au; 0.72 mg/kg) for 21 days. **n**, ChIP from liver lysates of wild-type mice using an Nrf2 antibody. Positive or negative controls were set as binding of Nrf2 to the *Nqo1* ARE or to a *Nqo1* ns region, respectively. Results are shown as percentage of input bound by the antibody. All data in plot represent mean ± SEM. Different letters indicate the significant difference ($P < 0.05$) analyzed by one-way ANOVA. n = 6. Source data are provided as a Source Data file.

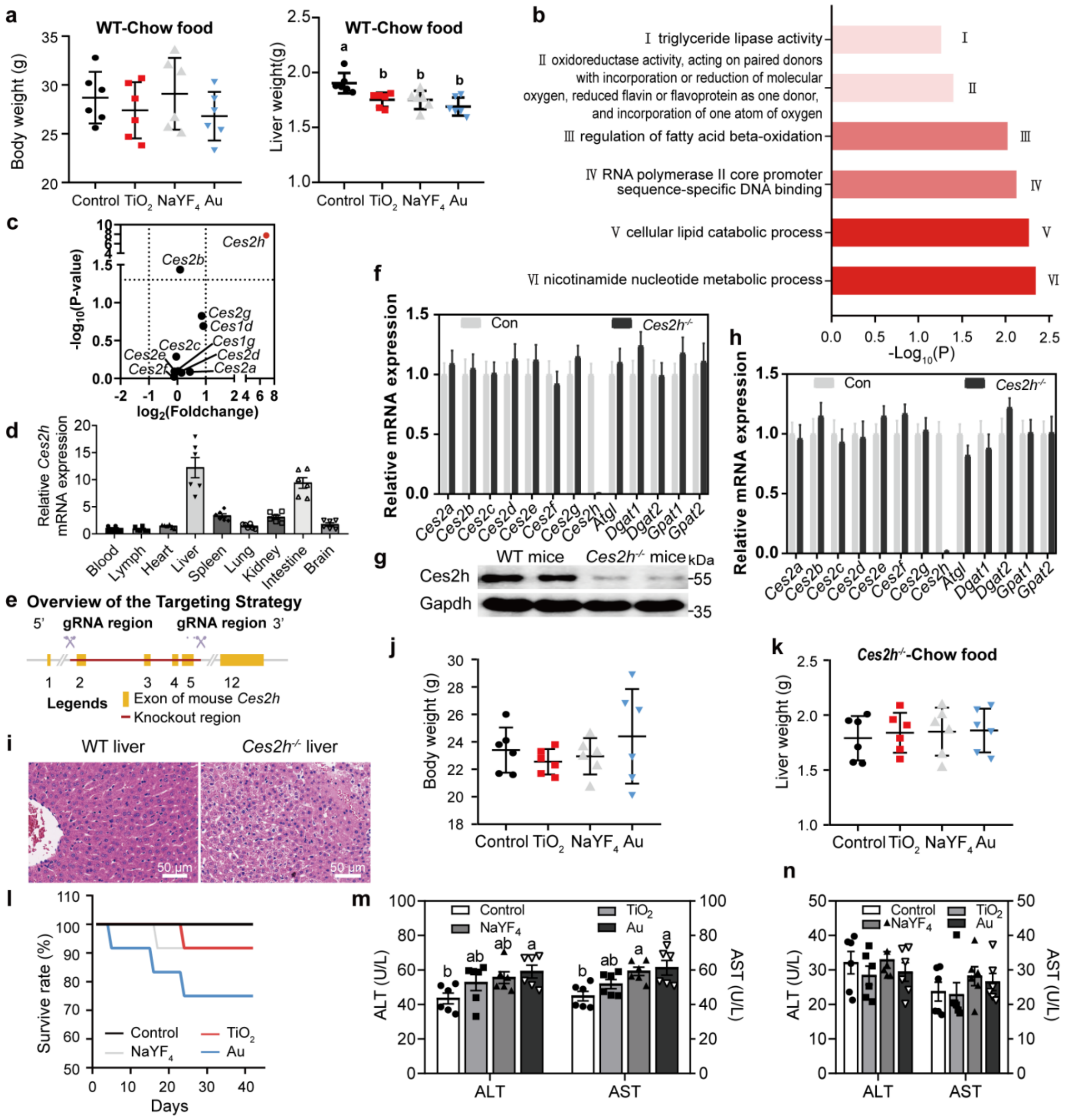

Supplementary Fig. 10 | Oral nanoparticle effects on body weight, *Ces2h* expression, hepatic metabolism, and survival in wild-type and *Ces2h*-deficient (*Ces2h*$^{-/-}$) mice. **a**, Effect of nanoparticles (0.72 mg/kg/day) on the body weight (left) and liver weight (right) in the wild-type mice (n = 6). **b**, Functional enrichment analysis of differential expressed genes from hepatic transcriptome comparison of TiO_2 group (0.72 mg/kg/day) vs control group. The differential expressed genes were defined by the $|log_2FC| > 1$ and $P < 0.05$. **c**, Changes in mRNA expression of the carboxylesterase family (*Ces1* and *Ces2*) in livers of wild-type mice treated with TiO_2 as determined by transcriptome. **d**, Relative mRNA expression of *Ces2h* in major organs of wild-type mice determined by RT-qPCR (n = 6). **e**, Overview of the targeting strategy to knockout *Ces2h* gene. **f**, Relative mRNA expression of *Ces2* family, canonical triglyceride lipase *Atgl*, and re-esterification pathways in livers of *Ces2h*$^{-/-}$ mice compared with wild-type mice (n = 6). **g**, Ces2h protein levels in liver of *Ces2h*$^{-/-}$ mice compared with wild-type mice. **h**, Relative mRNA expression of *Ces2* family, canonical triglyceride lipase *Atgl*,

and re-esterification pathways in intestines of *Ces2h*$^{-/-}$ mice compared with wild-type mice (n = 6). **i**, Histological analysis of *Ces2h*$^{-/-}$ mouse liver compared with wild-type mouse liver. **j**-**k**, Body and liver weight of *Ces2h*$^{-/-}$ mice fed with chow food and with/without oral nanoparticle administration (0.72 mg/kg/day; n = 6). **l**-**m**, Survivorship curve (n = 12) and serum liver index (alanine transaminase, ALT; aspartate aminotransferase, AST) (n = 6) of *Ces2h*$^{-/-}$ mice fed with chow food and with/without oral nanoparticle administration (0.72 mg/kg/day). **n**, Serum liver index (alanine transaminase, ALT; aspartate aminotransferase, AST) of wild-type mice fed with chow food and with/without oral nanoparticle administration (0.72 mg/kg/day; n = 6). Different letters indicate the significant difference ($P < 0.05$) analyzed by one-way ANOVA. Data in **a**, **d**, **f**, **h**, **j**, **k**, **m**, **and n** are presented as mean values ± SEM. Source data are provided as a Source Data file.

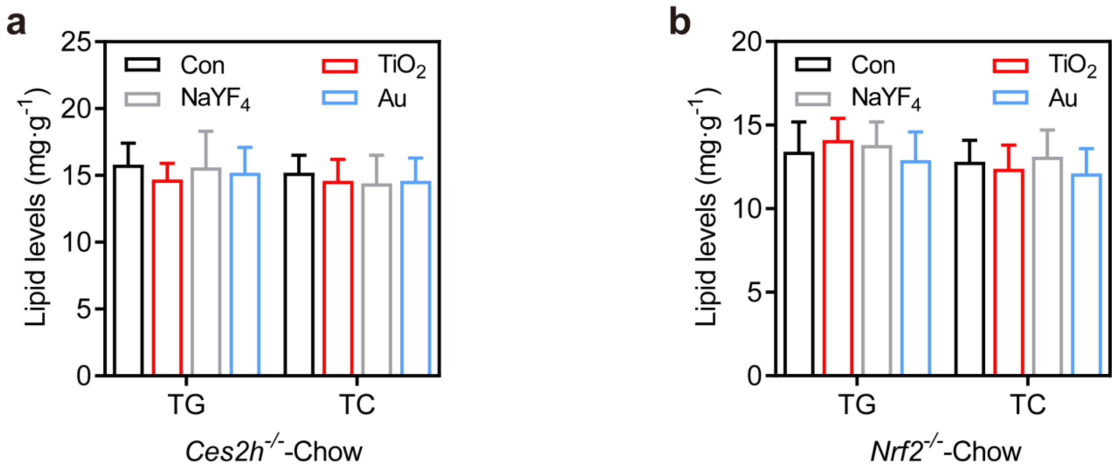

Supplementary Fig. 11 | Oral nanoparticle effects (0.72 mg/kg/day for 21 days) on hepatic lipid levels in *Ces2h*-deficient (a), *Nrf2*-deficient (b). TG: triglyceride; TC: total cholesterol. Data are presented as mean values ± SEM. n = 6. Source data are provided as a Source Data file.

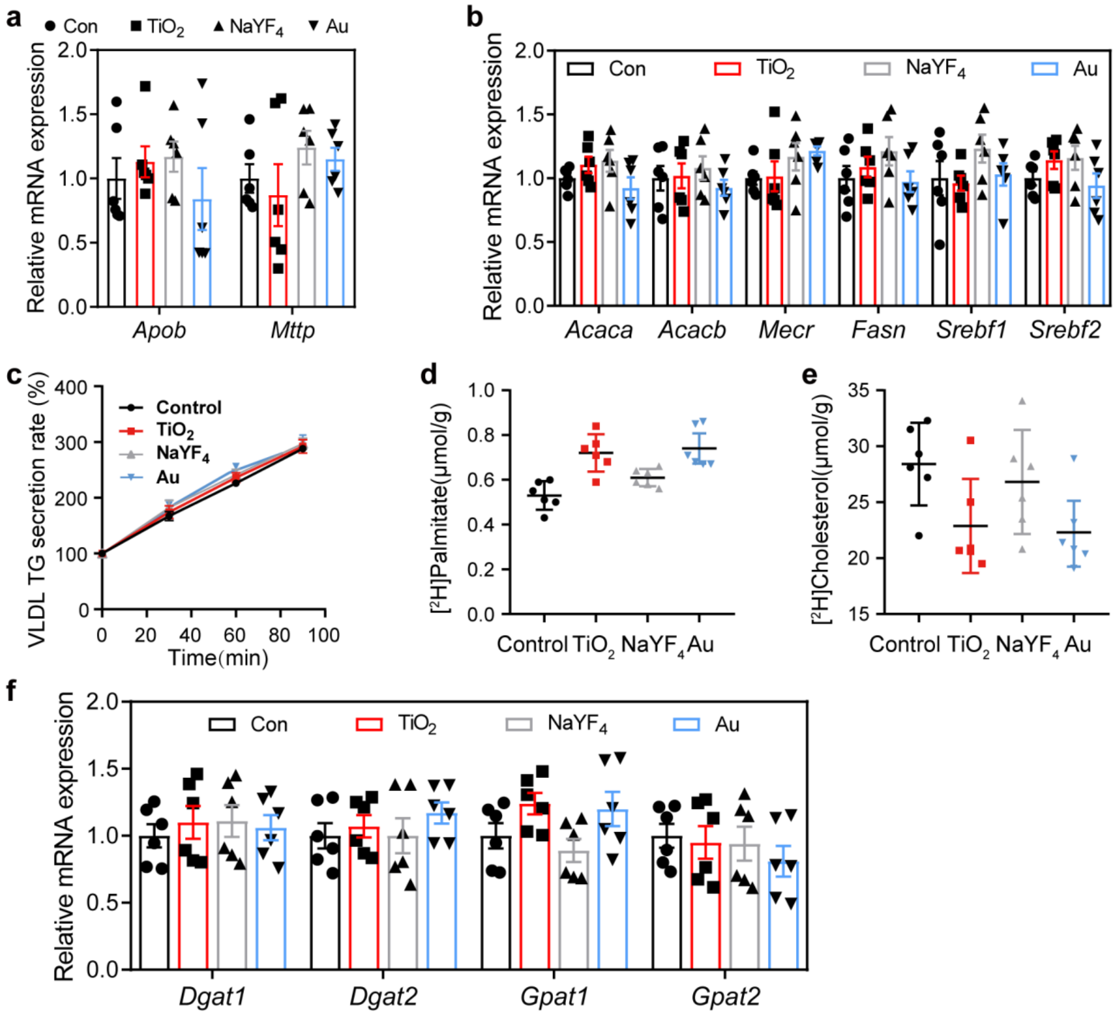

Supplementary Fig. 12 | Oral nanoparticle effects on hepatic lipid secretion, *de novo* lipogenesis, and re-esterification in wild-type mice. a, Hepatic mRNA levels of genes related to very low-density lipoprotein (VLDL) secretion influenced by nanoparticles (0.72 mg/kg/day for 21 days) measured by RT-qPCR. **b**, Hepatic mRNA levels of lipogenic genes influenced by nanoparticles (0.72 mg/kg/day for 21 days) measured by RT-qPCR in wild-type mice fed with chow food. **c**, VLDL secretion rate was measured in wild-type mice fed with chow food and with/without nanoparticle administration. **d**-**e**, *De novo* lipogenesis was evaluated in mice after injection of $^{2}H_2O$. The hepatic levels of newly synthesized [^{2}H]palmitate (**d**), or [^{2}H]cholesterol (**e**) were measured. **f**, Hepatic mRNA levels of re-esterification genes influenced by nanoparticles (0.72 mg/kg/day for 21 days) measured by RT-qPCR in wild-type mice fed with chow food. Data are presented as mean values ± SEM. n = 6. Source data are provided as a Source Data file.

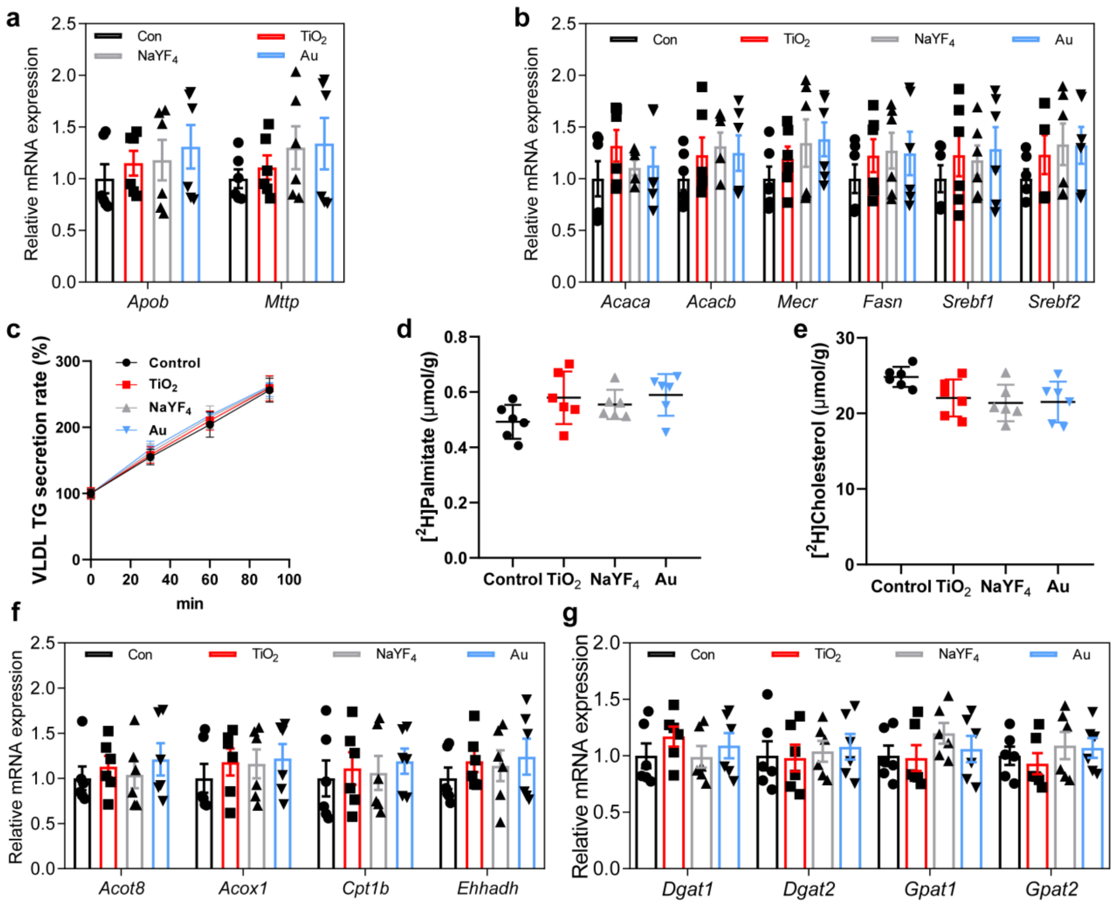

Supplementary Fig. 13 | Oral nanoparticle effects on hepatic lipid secretion, *de novo* lipogenesis, and re-esterification in *Nrf2*$^{-/-}$ mice. a, Hepatic mRNA levels of very low-density lipoprotein (VLDL) secretion related genes influenced by nanoparticles (0.72 mg/kg/day for 21 days) measured by RT-qPCR. **b**, Hepatic mRNA levels, measured by RT-qPCR, of lipogenic genes influenced by nanoparticles (0.72 mg/kg/day for 21 days) in *Nrf2*$^{-/-}$ mice fed with chow food. **c**, VLDL secretion rate was measured in the *Nrf2*$^{-/-}$ mice fed with chow food and with/without nanoparticle administration. **d-e**, *De novo* lipogenesis was evaluated in mice after injection of ^2H_2O. The hepatic levels of newly synthesized [^{2}H]palmitate (**d**), or [^{2}H]cholesterol (**e**). **f-g**, Hepatic mRNA levels, measured by RT-qPCR, of fatty acid oxidation genes, and re-esterification genes influenced by nanoparticles (0.72 mg/kg/day for 21 days) in *Nrf2*$^{-/-}$ mice fed with chow food. Data are presented as mean values ± SEM. n = 6. Source data are provided as a Source Data file.

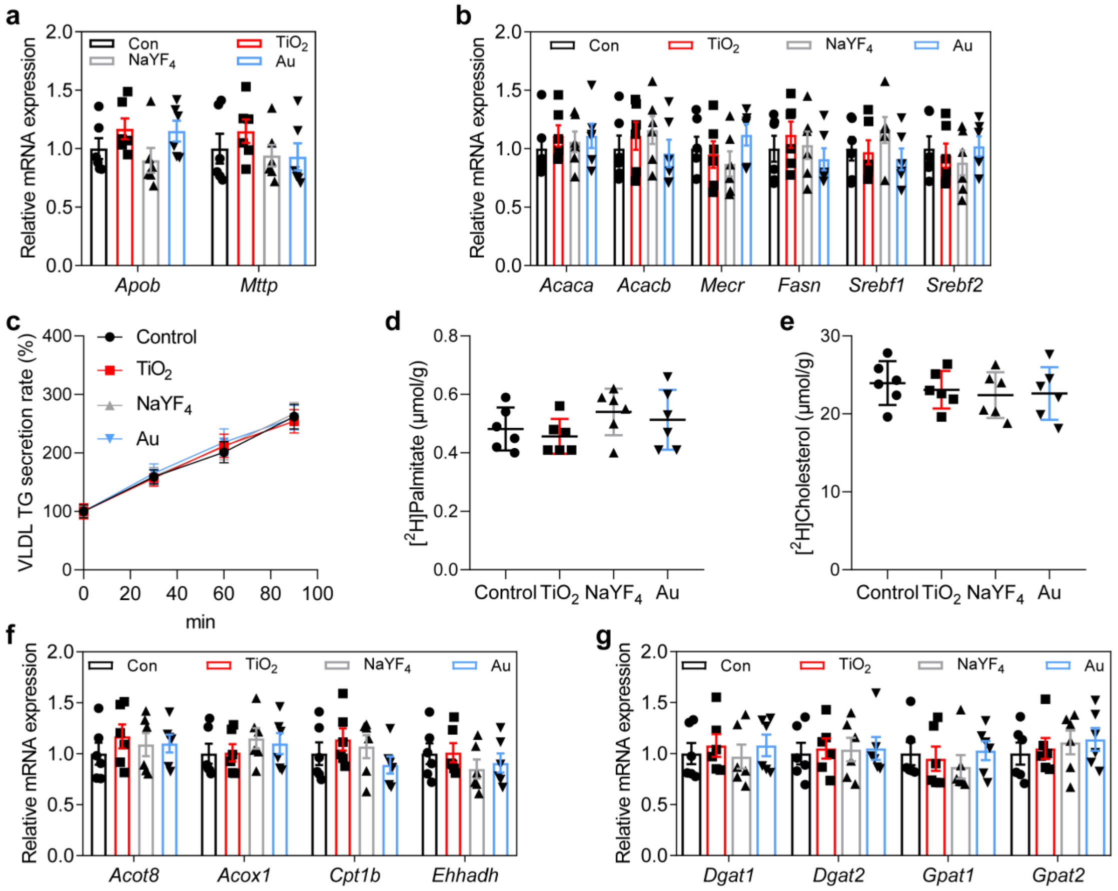

Supplementary Fig. 14 | Oral nanoparticle effects on hepatic lipid secretion, *de novo* lipogenesis, and re-esterification in *Ces2h*$^{-/-}$ mice. a, Hepatic mRNA levels, measured by RT-qPCR, of very low-density lipoprotein (VLDL) secretion related genes influenced by nanoparticles (0.72 mg/kg/day for 21 days). **b**, Hepatic mRNA levels, measured by RT-qPCR, of lipogenic genes influenced by nanoparticles (0.72 mg/kg/day for 21 days) in *Ces2h*$^{-/-}$ mice fed with chow food. **c**, VLDL secretion rate was measured in the *Ces2h*$^{-/-}$ mice fed with chow food and with/without nanoparticle administration. **d-e**, *De novo* lipogenesis was evaluated in mice after injection of ^2H_2O. The hepatic levels of newly synthesized [^{2}H]palmitate (**d**), or [^{2}H]cholesterol (**e**) were measured. **f-g**, Hepatic mRNA levels, measured by RT-qPCR, of fatty acid oxidation genes, and re-esterification genes influenced by nanoparticles (0.72 mg/kg/day for 21 days) in *Ces2h*$^{-/-}$ mice fed with chow food. Data are presented as mean values ± SEM. n = 6. Source data are provided as a Source Data file.

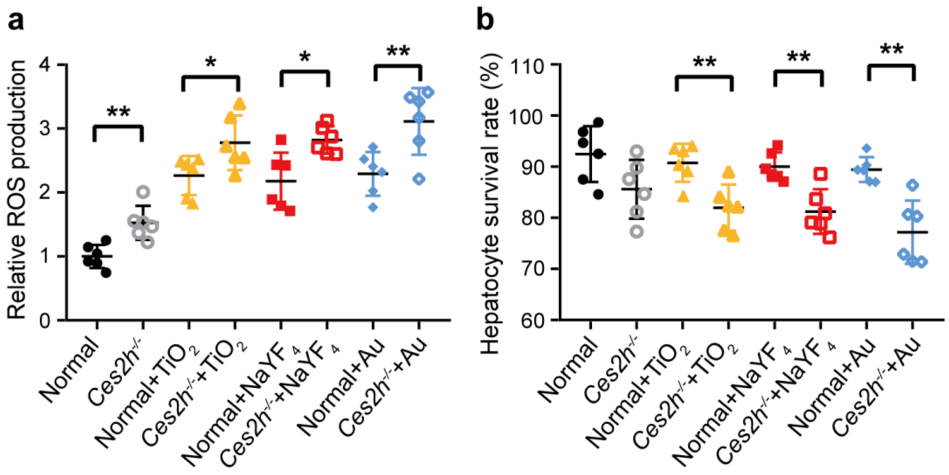

Supplementary Fig. 15 | Different effects of nanoparticles on cellular ROS production, and cell survival rate between normal or *Ces2h*-deficient (*Ces2h*$^{-/-}$) hepatocytes. The normal or *Ces2h*$^{-/-}$ mice were treated with either saline or nanoparticles (0.72 mg/kg/day) by gavage. The hepatocytes were identified according to the gating strategy presented in the Fig. S7. Two-sided student's t-test was applied to ROS production (**a**) and hepatocyte survival rate (**b**) with a significance define as followed: *, $P < 0.05$; **, $P < 0.01$. Different letters indicate the significant difference ($P < 0.05$). Data are presented as mean values ± SEM. n = 6. Source data are provided as a Source Data file.

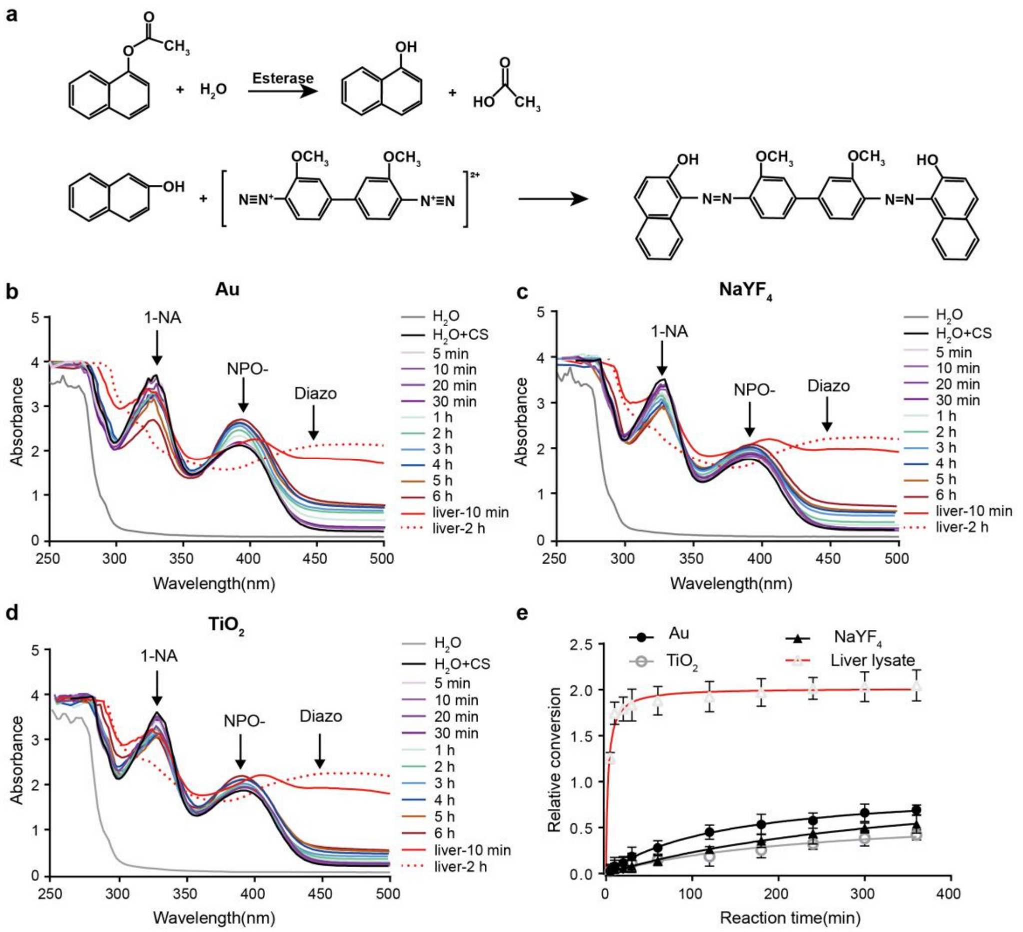

Supplementary Fig. 16 | Chemical catalysis of esters by nanoparticles. a, Diagram illustrating the measurement of esterase activity. **b-d**, Absorbance spectra of reaction supernatants sampled at selected time intervals. The reaction was catalyzed by of Au, $NaYF_4$, TiO_2 and positive control (liver lysate) at 37°C. The characteristic absorption peak was observed near 320 nm for 1-Naphthyl acetate (1-NA), near 390 nm for intermediate naphthol peak and near 450 nm for final product diazo. **e**, The relative conversion of 1-NA to diazo was determined for esterase activity of nanoparticles and positive control (liver lysate) at 37°C. Data in **e** are presented as mean values ± SEM. n = 6.

a

Protein	Ces2h	CES2	CES1
Similarity	48.05%	49.60%	—

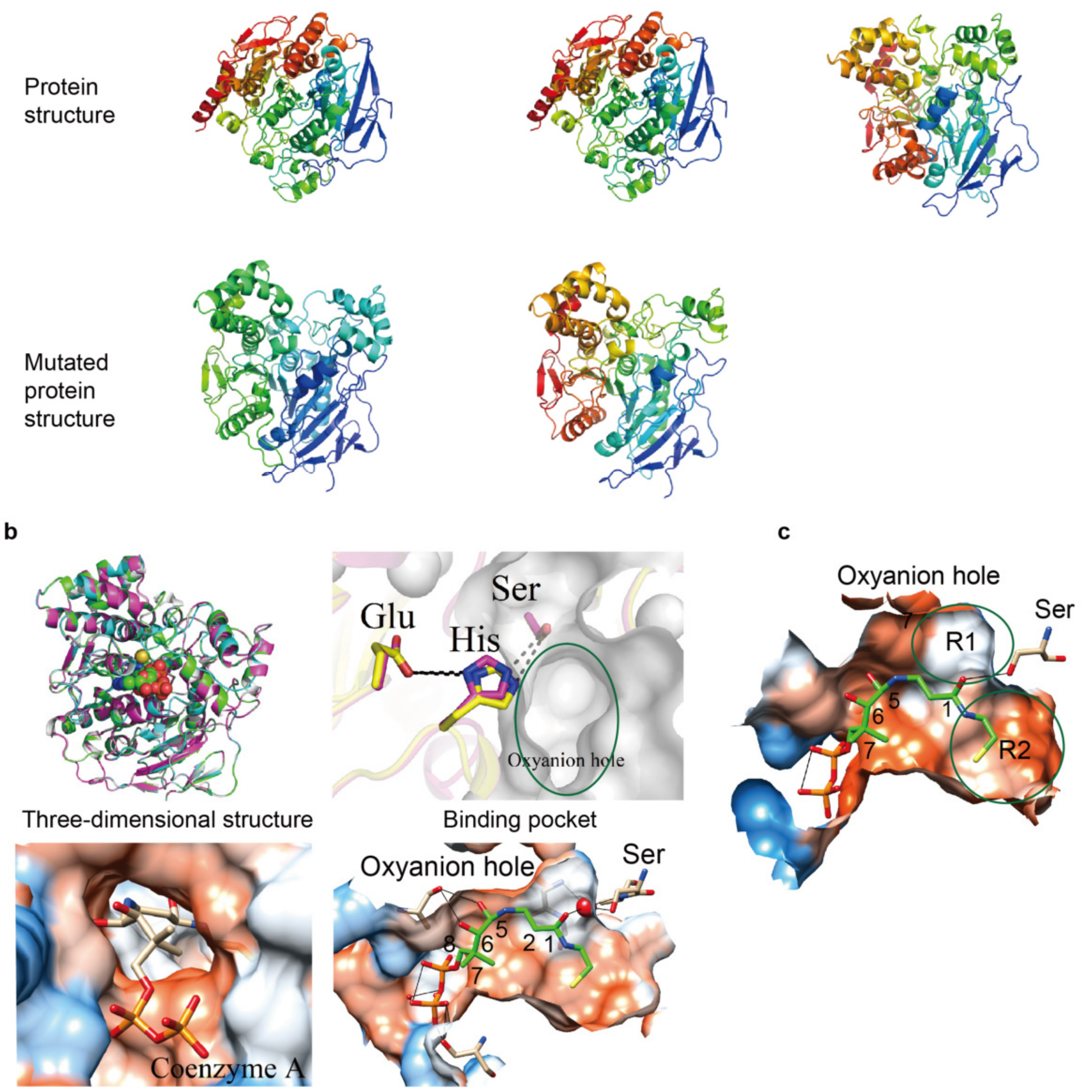

Supplementary Fig. 17 | Homologous modelling and optimization of *Homo sapiens* and *Mus musculus* carboxylesterase 2 (CES2/Ces2h). a, Crystal structures of CES2, Ces2h, point-mutated CES2 (HG^{192}G^{193}A> HG^{192}A^{193}A), point-mutated Ces2h (HG^{147}G^{148}S> HG^{147}A^{148}S), and the protein template CES1. The similarity between Ces2h and CES1, CES2 and CES1 was 48.05% and 49.60%, respectively. **b**, Protein structure and pocket structure obtained after optimization of molecular dynamics. The results showed that the structures of CES1, CES2, and Ces2h were almost identical, with no significant differences, especially near the binding pockets. **c**, Pocket identification and analysis based on a similar crystal structure. The small molecule is endogenous with its end inserted into the hydrophobic pocket. C5, C6, C7, and C8 of the molecule all contain branched chains, indicating that this part of the pocket is suitable for molecules of similar or smaller size. The end of C8 corresponds to the

phosphate molecule, which is surrounded by polar pockets. If pure hydrophobic molecules are connected to this position, the peripheral region of C8 cannot be connected, because this region is hydrophilic. R1 region: Oxyanion hole region; R2 region: Pocket position binding with the hydrophobic carbon chain in the deep pocket; 1-2-3-4-5-6-7-8: All the locations are hydrophobic pockets, which will be an important binding region for the carbon chain group of cholesterol ester. Our molecule will move around in this region, as evident in our following docking system.

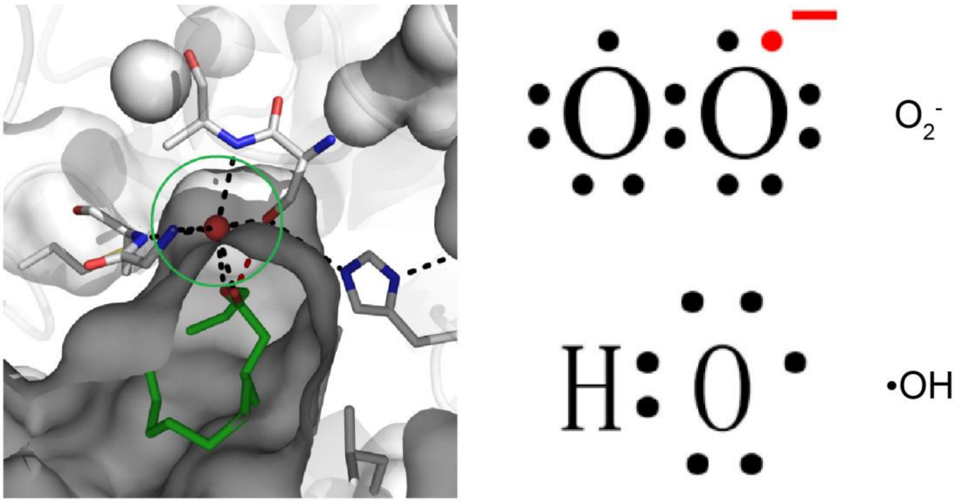

Supplementary Fig. 18 | Docking analysis of molecular systems of *Homo sapiens* and *Mus musculus* carboxylesterase 2 (CES2/Ces2h)-superoxide anion (O_2^-)/hydroxyl radical (·OH)-ester. In the docking analysis, O_2^- and ·OH was added at the same time, and 500 ps of non-limiting molecular dynamics analysis was performed using Amber 16. Kinetic constraint was set as followed: 1. ester, O_2^-, and ·OH was unrestricted; 2. Docking analysis was completely flexible; 3. Proteins were kept rigid. The average structure was extracted from the trajectory of molecular dynamics and used for subsequent analysis. Preliminary optimization results of dynamics were as followed: 1. Due to the limited space of oxygen anion hole, the co-existence of O_2^- and ·OH in the protein-molecule docking complex was not detected; 2. In the CES2 protein system, O_2^- was not located at the oxyanion hole, but ·OH stayed at this position; 3. Similarly in the Ces2h protein system, O_2^- was not located at oxyanion hole, but ·OH stayed at this position and formed a hydrogen bond interaction with the ligand.

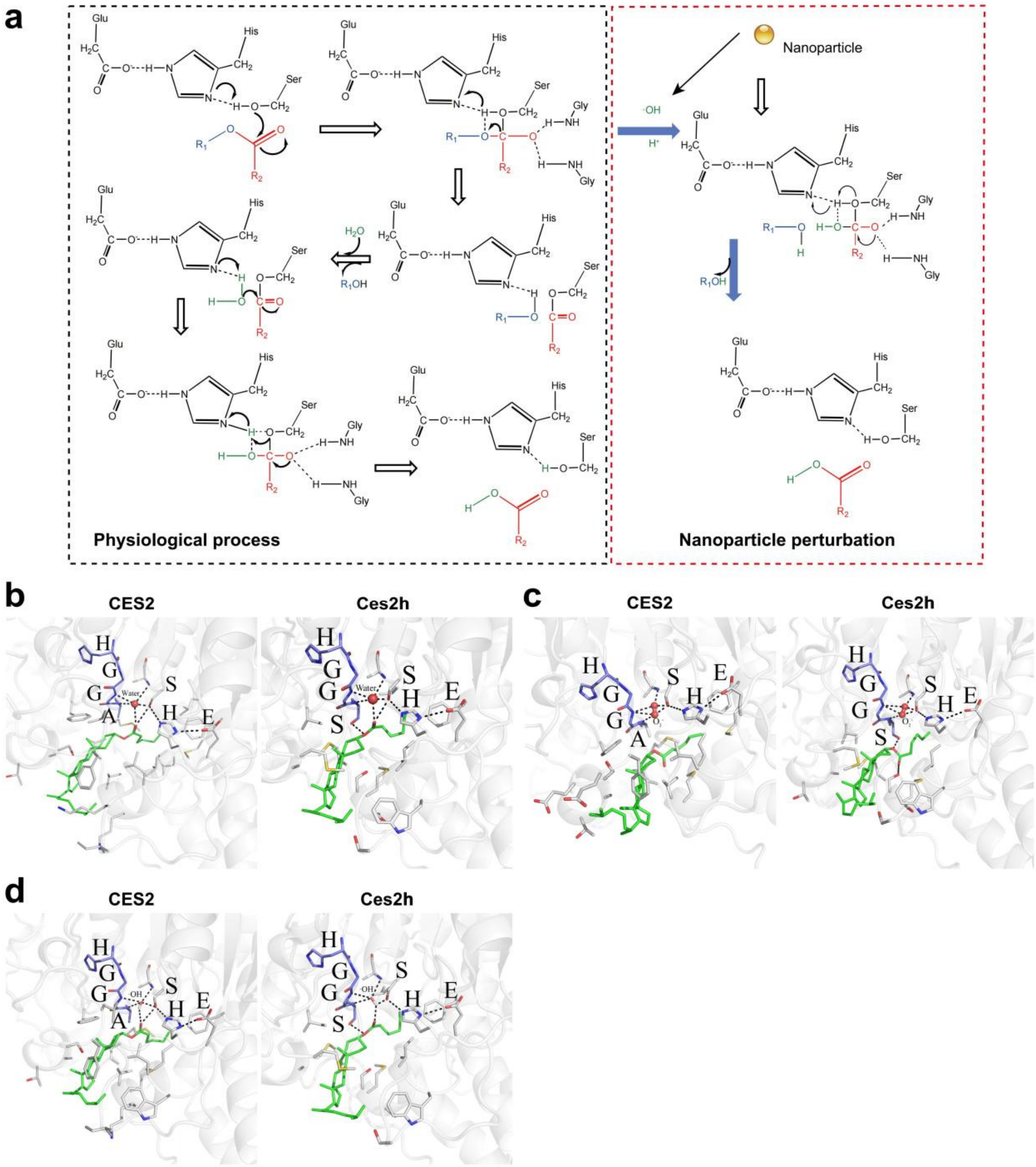

Supplementary Fig. 19 | Molecular modeling and docking of *Homo sapiens* and *Mus musculus* carboxylesterase 2 (CES2/Ces2h) protein with reactive oxygen species induced by nanoparticles. a, The schematic diagram of catalytic mechanism of carboxylesterases and the perturbation of nanoparticles. **b**, Molecular docking of CES2/Ces2h with ester. Blue stick: oxyanion hole; Green stick: ligand; Black dash: hydrogen-bond interaction. **c**, Molecular docking of CES2/Ces2h with ester and $\cdot O_2^-$. Blue stick: oxyanion hole; Green stick: ligand; Black dash: hydrogen-bond interaction. **d**, Molecular docking of CES2/Ces2h with ester and ·OH. Blue stick: oxyanion hole; Green stick: ligand; Black dash: hydrogen-bond interaction.

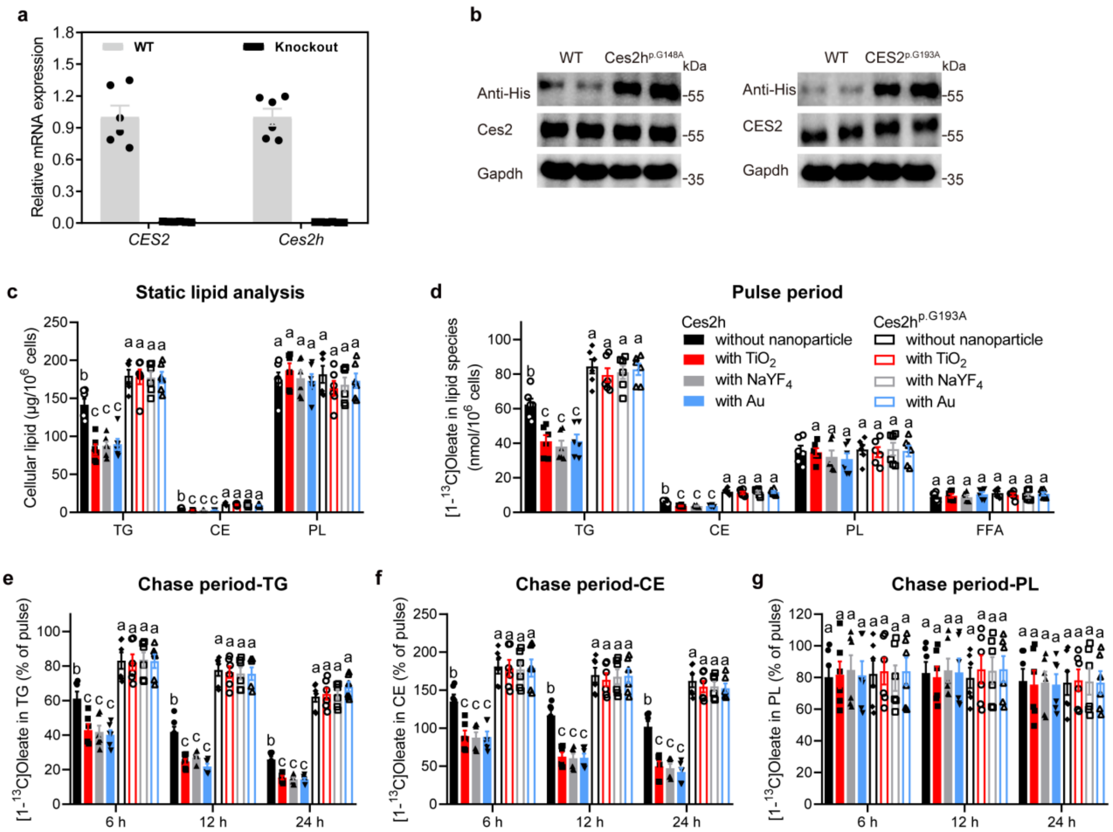

Supplementary Fig. 20 | Immunofluorescence staining showing catalytic ability of hepatocytes (n = 6) with normal CES2 or mutated CES2$^{p.G193A}$ on ester and the perturbation of nanoparticles. a, Knockout effects of *CES2* and *Ces2h* in LO2 and NCTC1469 cell lines, respectively. **b**, Ces2h$^{p.G148A}$ and CES2$^{p.G193A}$ expression after transfecting LO2/NCTC1469 cells with the corresponding eukaryotic expression vectors (pcDNA3.1(+)-His-*CES2*$^{g.695G>C}$, pcDNA3.1(+)-His-*Ces2h*$^{g.436G>C}$) and their protein level of Ces2h/CES2 compared to their wild-type cells. **c**, Intracellular lipid fraction analysis of normal CES2 or mutated CES2$^{p.G193A}$ hepatocyte cell lines upon TiO_2, $NaYF_4$, Au nanoparticle treatments, respectively. **d**, Incorporation of [1-^{13}C]oleate into cellular lipids. **e-g**, Chase experiments evaluating turnover of lipid species, including triglyceride (TG), cholesterol ester (CE), and phospholipid (PL). Statistics for the chase period were analyzed as a percentage of the pulse. The group setting was same in (**c-g**) as indicated in (**d**). Different letters indicate the significant difference ($P < 0.05$) analyzed by one-way ANOVA. Data in **a**, **c-g** are presented as mean values ± SEM. n = 6. Source data are provided as a Source Data file.

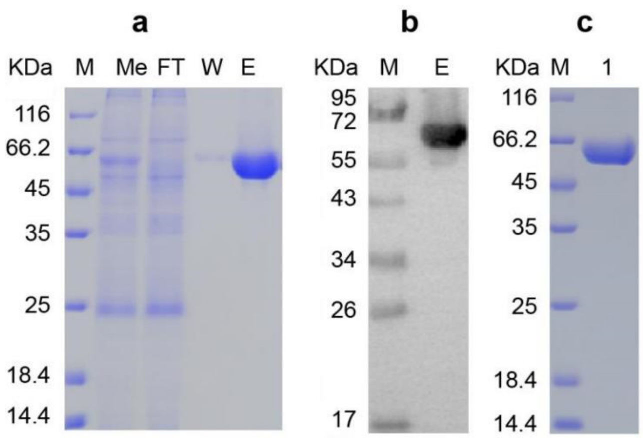

Supplementary Fig. 21 | Mouse carboxylesterase 2h (Ces2h) protein purification profile. a, Coomassie blue staining of purified Ces2h. M, Molecular weight marker. Me, Culture medium. FT, Flow through. W, Washes. E, Eluted fractions. **b**, Western blotting, anti-His detection and ECL revelation. M, Molecular weight marker. E, Eluted fractions. **c**, Final sample QC of Ces2h by Coomassie blue staining. M, Molecular weight marker. 1, Ces2h sample.

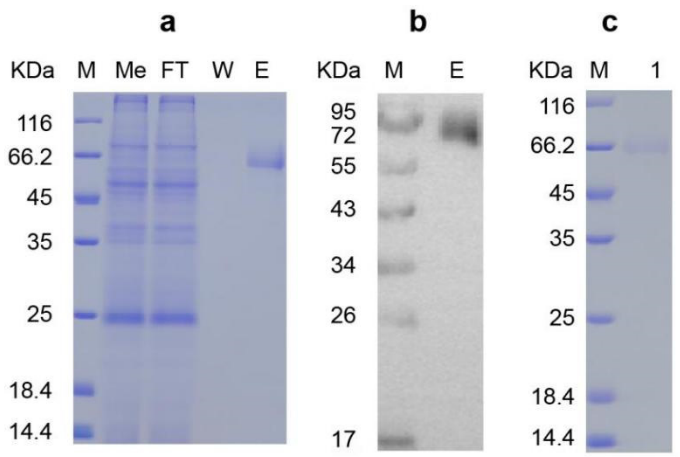

Supplementary Fig. 22 | Human carboxylesterase 2 (CES2) protein purification profile. a, Coomassie blue staining of purified CES2. M, Molecular weight marker. Me, Culture medium. FT, Flow through. W, Washes. E, Eluted fractions. **b**, Western blotting, anti-His detection and ECL revelation. M, Molecular weight marker. E, Eluted fractions. **c**, Final sample QC of CES2 by Coomassie blue staining. M, Molecular weight marker. 1, CES2 sample.

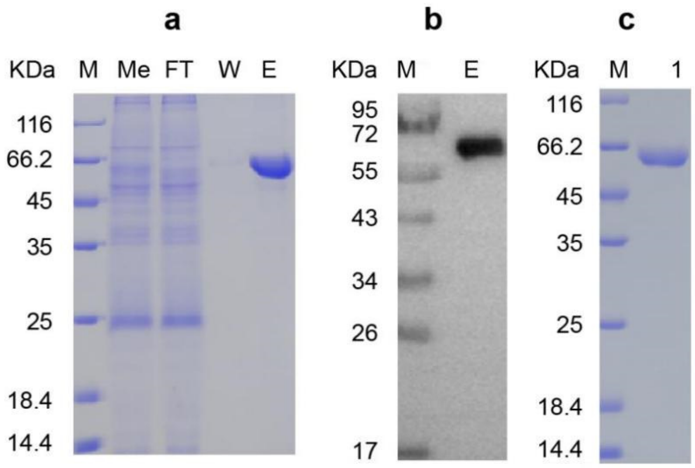

Supplementary Fig. 23 | Mutated mouse carboxylesterase 2h (Ces2h$^{p.G148A}$) protein purification profile. a, Coomassie blue staining of purified Ces2h$^{p.G148A}$. M, Molecular weight marker. Me, Culture medium. FT, Flow through. W, Washes. E, Eluted fractions. **b**, Western blotting, anti-His detection and ECL revelation. M, Molecular weight marker. E, Eluted fractions. **c**, Final sample QC of Ces2h$^{p.G148A}$ by Coomassie blue staining. M, Molecular weight marker. 1, Ces2h$^{p.G148A}$ sample.

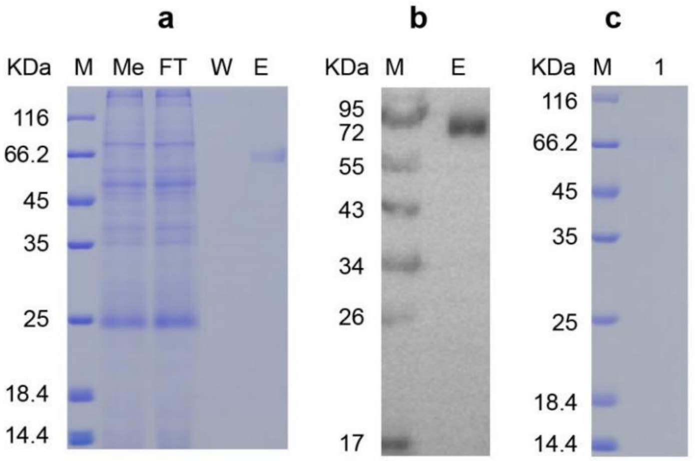

Supplementary Fig. 24 | Mutated human carboxylesterase 2 (CES2$^{p.G193A}$) protein purification profile. a, Coomassie blue staining of purified CES2$^{p.G193A}$. M, Molecular weight marker. Me, Culture medium. FT, Flow through. W, Washes. E, Eluted fractions. **b**, Western blotting, anti-His detection and ECL revelation. M, Molecular weight marker. E, Eluted fractions. **c**, Final sample QC of CES2$^{p.G193A}$ by Coomassie blue staining. M, Molecular weight marker. 1, CES2$^{p.G193A}$ sample.

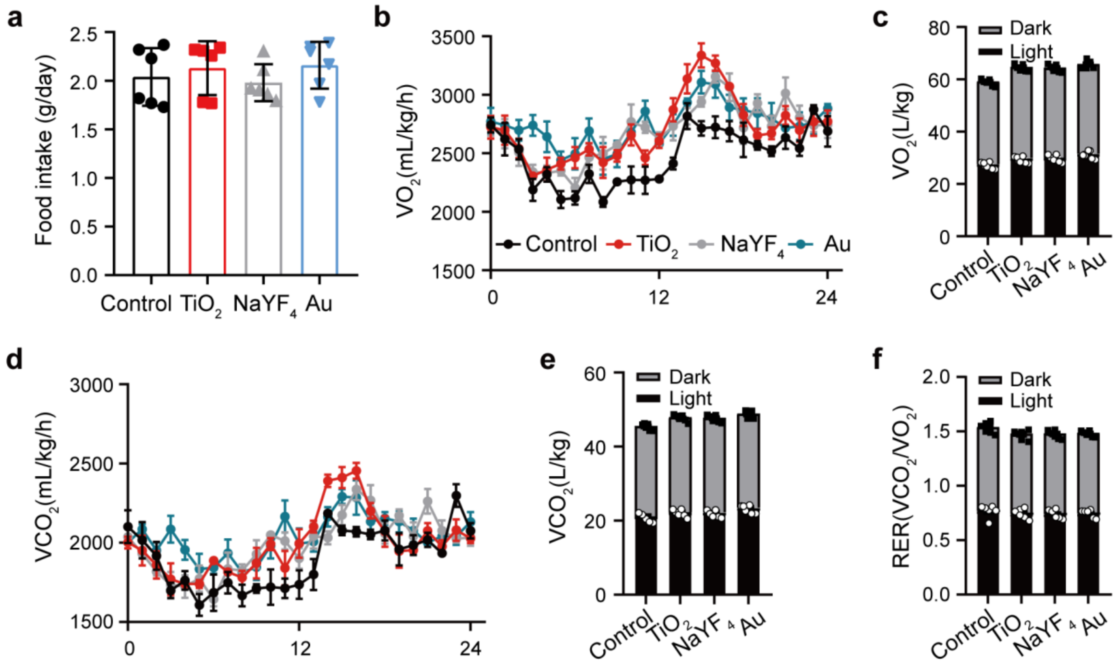

Supplementary Fig. 25 | Oral nanoparticle effects on whole-body energy metabolic activity. a, Food intake of *db/db* mice fed with chow food with or without oral nanoparticle treatment (0.72 mg/kg per dose). CLAMS was used to determine 24-hour O2 consumption (**b**-**c**), CO2 production (**d**-**e**), and respiration quotient rate (RER) (**f**). Different letters indicate the significant difference ($P < 0.05$) analyzed by one-way ANOVA. Data in **a**-**f** are presented as mean values ± SEM. n = 6. Source data are provided as a Source Data file.

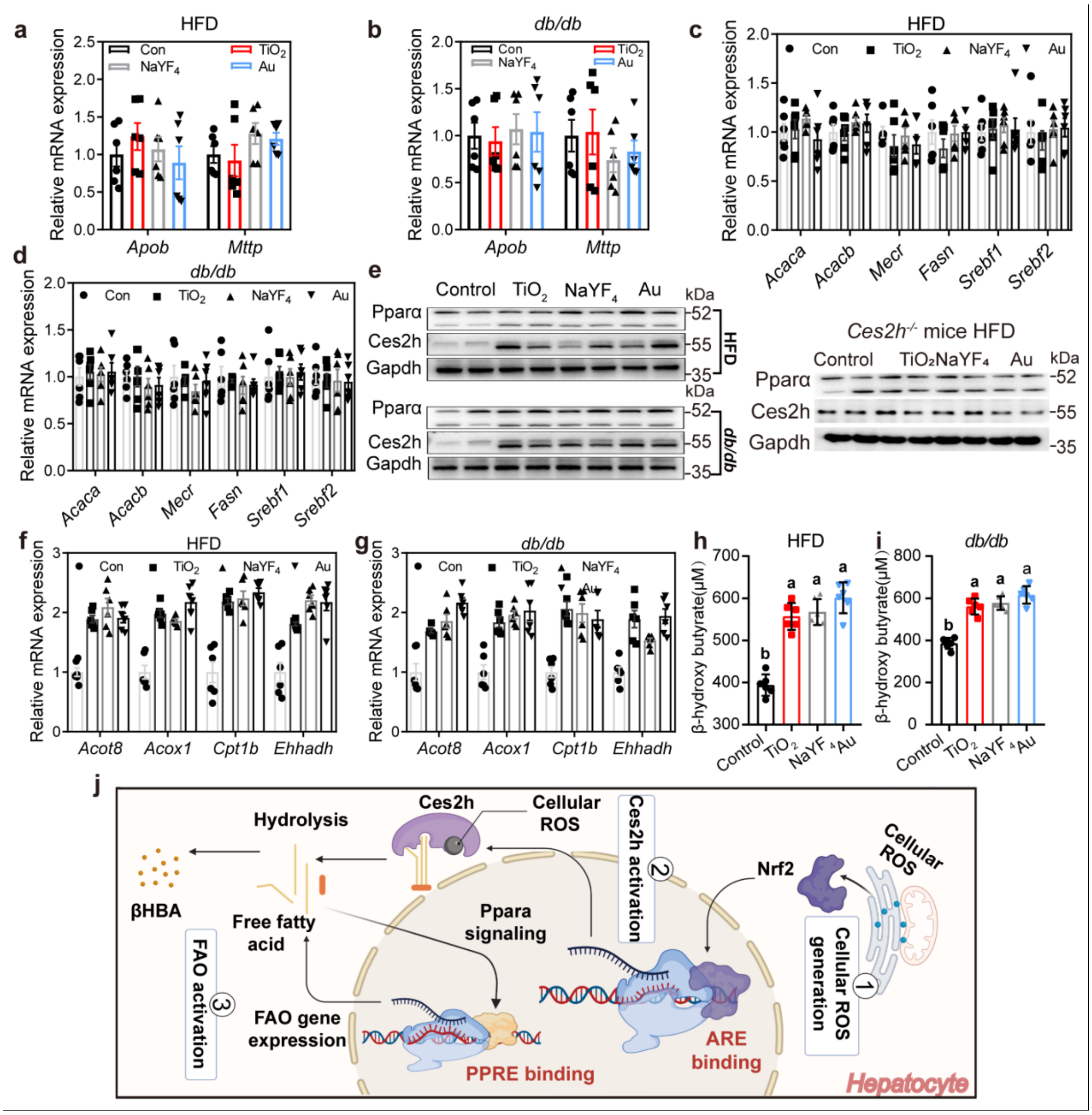

Supplementary Fig. 26 | Nanoparticle-activated *Ces2h* expression and its downstream fatty acid oxidation (FAO) promote the hepatic lipid metabolism in high fat diet (HFD) and genetically obese (*db/db*) mice. Hepatic mRNA levels, measured by RT-qPCR, of very low-density lipoprotein (VLDL) secretion related genes influenced by nanoparticles (0.72 mg/kg per dose) in HFD mice (**a**) and *db/db* mice (**b**). Hepatic mRNA levels, measured by RT-qPCR, of lipogenic genes influenced by nanoparticles in HFD mice (**c**), and *db/db* mice (**d**). **e**, Hepatic protein expression of Pparα and Ces2h in HFD mice (left top), *db/db* mice (left bottom), and *Ces2h*$^{-/-}$ mice fed with HFD (right). The experiment was repeated for 3 times, and the figure shown is the representative results. Hepatic mRNA levels, measured by RT-qPCR, of FAO genes in HFD mice (**f**), and *db/db* mice (**g**). Plasma β-hydroxybutyrate (βHBA) level in HFD mice (**h**), and *db/db* mice (**i**). **j**, The signaling pathway of nanoparticle-perturbed lipid metabolism in liver. Nanoparticles induced the expression of *Ces2h* and simultaneously facilitated Ces2h to hydrolyze triglycerides. The derived free fatty acid functioned as a ligand to augment Pparα activity. As a result, FAO genes under the

regulation of Pparα was upregulated and catalyzed the free fatty acid. Different letters indicate the significant difference ($P < 0.05$) analyzed by one-way ANOVA. Data in **a**-**d**, **f**-**h**, are presented as mean values ± SEM. n = 6. Source data are provided as a Source Data file.

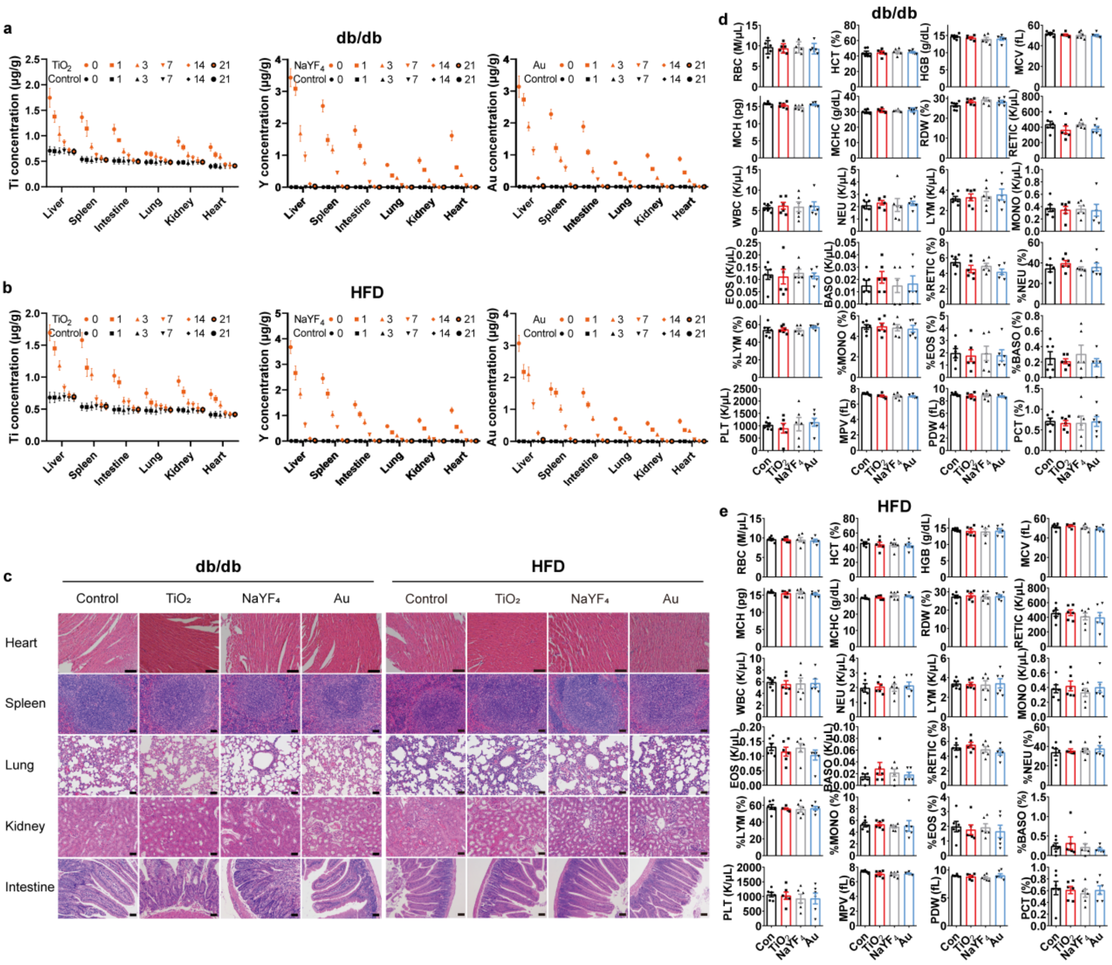

Supplementary Fig. 27 | Safety assessment of orally administrated nanoparticles in genetically obesity (*db/db*) mice and high fat diet (HFD)-fed mice. a-b, Clearance of orally administrated TiO_2, $NaYF_4$, and Au nanoparticles (0.72 mg/kg/day) for each organ in *db/db* mice (**a**) and HFD-fed C57BL/6 wild type mice (**b**). The oral administration procedure for nanoparticle treatments was performed every two days (46 times in total) during the 3-month treatment. The clearance data was collected 21 days after administration was stopped. **c**, The pathological analysis of the main organs in *db/db* mice and HFD-fed C57BL/6 wild-type mice with 3-month orally administrated TiO_2, $NaYF_4$, and Au nanoparticles (0.72 mg/kg/day) using HE staining. Scale bar, 100 µm. **d-e**, Hematology analysis of *db/db* mice (d) and HFD-fed C57BL/6 wild-type mice with 3-month orally administrated TiO_2, $NaYF_4$, and Au nanoparticles (0.72 mg/kg/day). All data in plot represent mean ± SEM. n = 6. Source data are provided as a Source Data file.

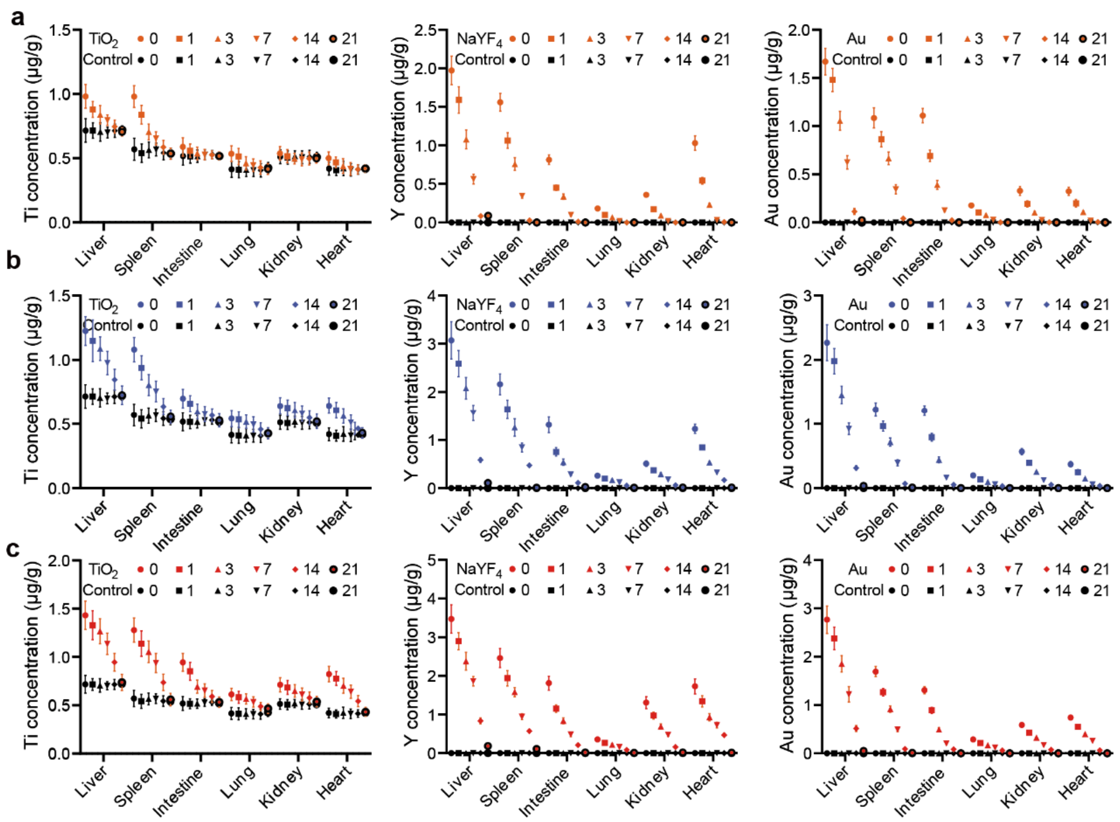

Supplementary Fig. 28 | Clearance of TiO_2, $NaYF_4$, and Au nanoparticles after 3-week low (a), middle (b), and high (c) oral administration for each organ. Day 0 represented the time when oral administration was stopped. All data in plot represent mean ± SEM. n = 6. Source data are provided as a Source Data file.

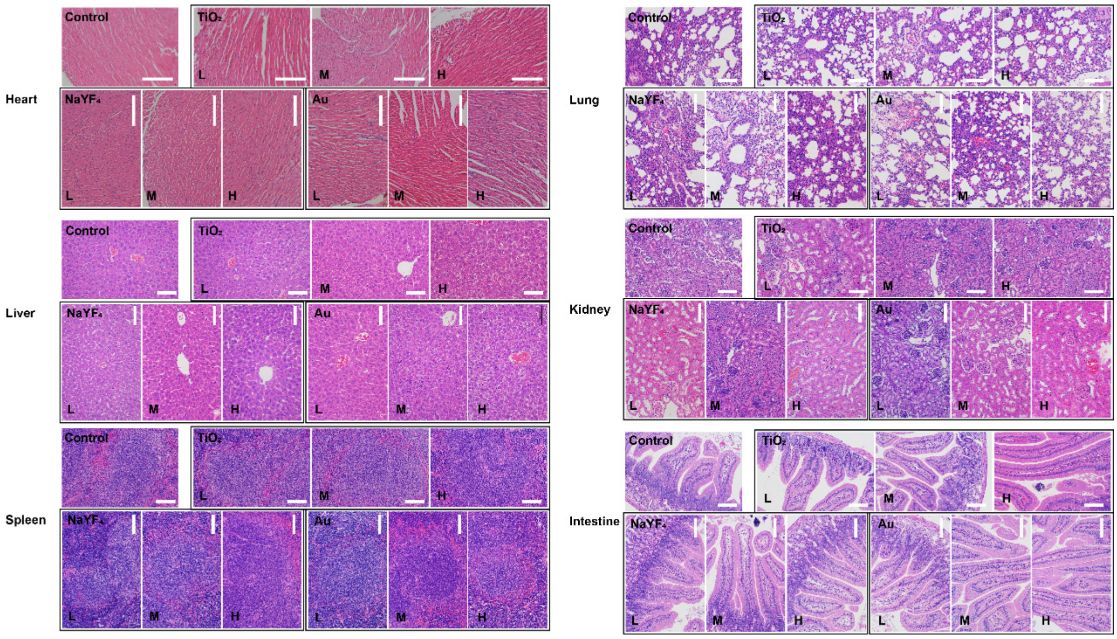

Supplementary Fig. 29 | Safety assessment of orally administrated nanoparticles with different doses. HE staining of main organs in mice with oral administrated TiO_2, $NaYF_4$, and Au nanoparticles (L, low dose, 0.72 mg/kg/day; M, middle dose, 1.8 mg/kg/day; H, high dose, 18 mg/kg/day) was performed on day 21 when oral administration was stopped. Scale bar, 100 μm.

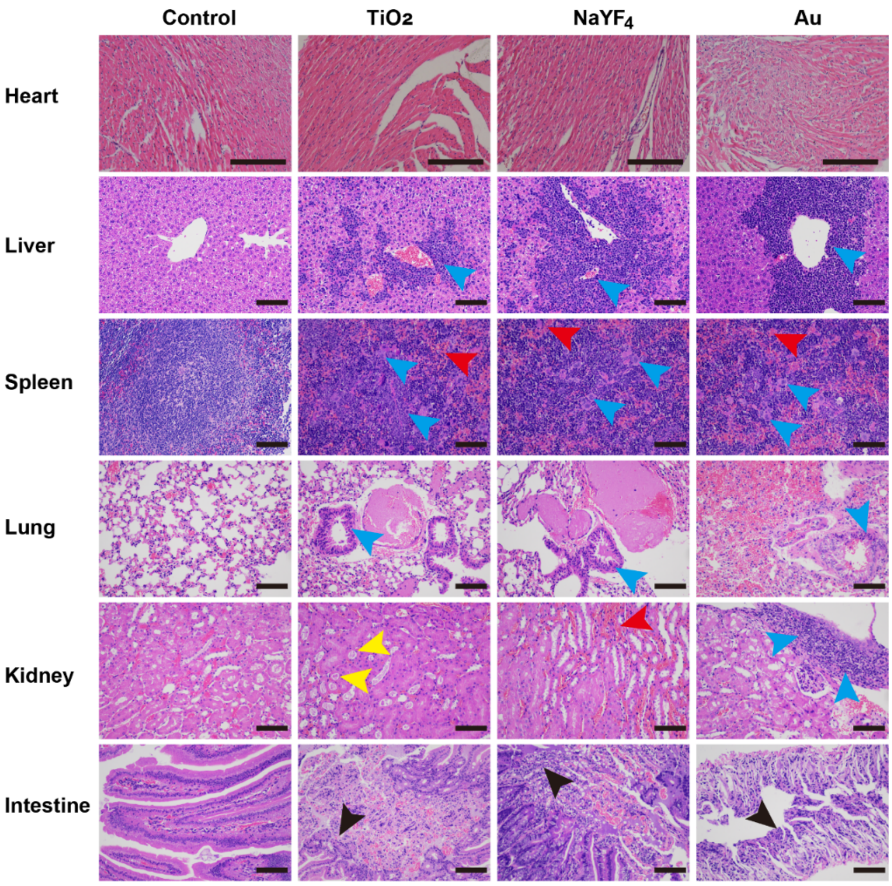

Supplementary Fig. 30 | Safety assessment of orally administrated nanoparticles in *Ces2h*-dificient (*Ces2h*$^{-/-}$) mice. HE staining of main organs in *Ces2h*$^{-/-}$ mice with/without oral administrated TiO_2, $NaYF_4$, and Au nanoparticles (0.72 mg/kg/day) was performed on day 21. Blue arrows indicate immune cell infiltrations; Red cells indicate aggregation of extramedullary hematopoietic cells in the spleen and tubulointerstitial congestion in the kidney; Yellow arrows indicate eosinophilic substances in the renal tubule lumen; Black arrows indicate bare lamina propria. Scale bar, 100 μm.

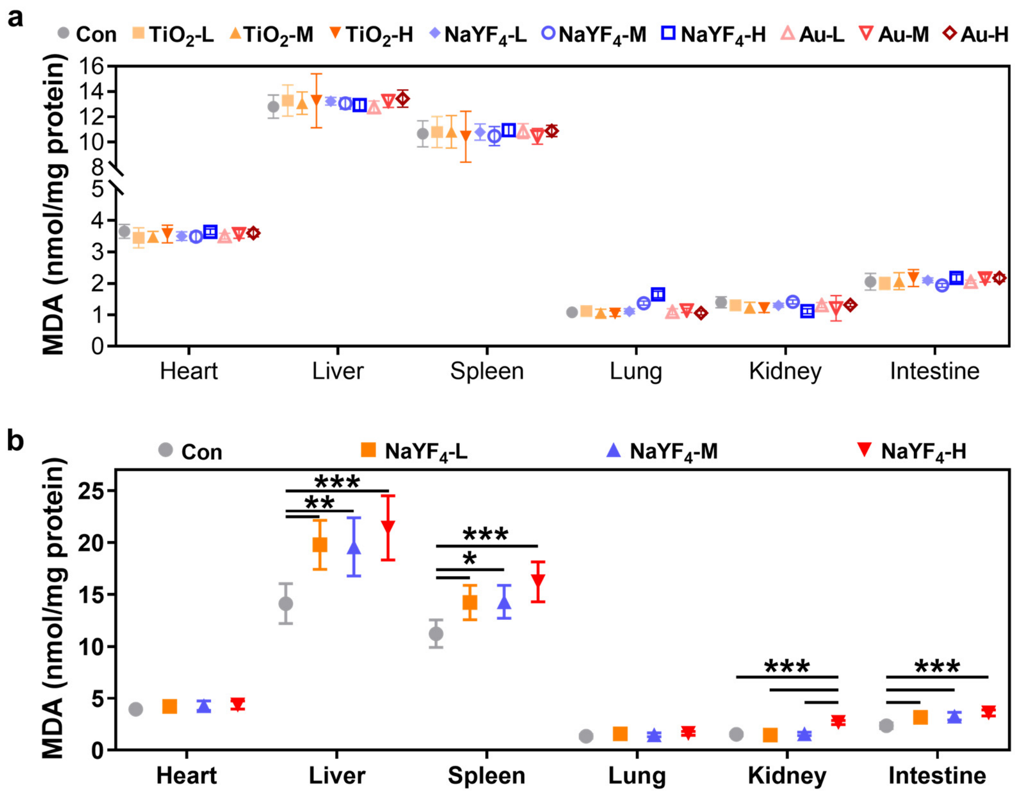

Supplementary Fig. 31 | Oxidative stress assessment of orally administrated nanoparticles in wild-type mice and *Ces2h*-dificient (*Ces2h*$^{-/-}$) mice. a, Malondialdehyde (MDA, product of oxidative stress) concentration in each tissue of wild-type mice with/without orally administrated TiO_2, $NaYF_4$, and Au (0.72 mg/kg/day) nanoparticles. Tissue samples were collected from parts of mice in each group on day 21. **b**, MDA concentration in each tissue of *Ces2h*$^{-/-}$ mice with/without orally administrated TiO_2, $NaYF_4$, and Au (0.72 mg/kg/day) nanoparticles. Tissue samples were collected from parts of mice in each group on day 21. * ($P < 0.05$), ** ($P < 0.01$), *** ($P < 0.001$) indicate the significant difference analyzed by one-way ANOVA. Data in **a**-**b** are presented as mean values ± SEM. n = 6. Source data are provided as a Source Data file.
