# Supplementary material for: Antioxidant hepatic lipid metabolism can be promoted by orally administered inorganic nanoparticles
Source: Nat Commun. 2023 Jun 20;14:3643. doi: 10.1038/s41467-023-39423-3 (PMC10281969; doi:10.1038/s41467-023-39423-3)
Supplement: Supplementary file 2 — Reporting Summary [file 41467_2023_39423_MOESM2_ESM.pdf]

nature portfolio

Corresponding author(s): Renren Deng

Last updated by author(s): May 26, 2023

Reporting Summary

Nature Portfolio wishes to improve the reproducibility of the work that we publish. This form provides structure for consistency and transparency in reporting. For further information on Nature Portfolio policies, see our Editorial Policies and the Editorial Policy Checklist.

Statistics

For all statistical analyses, confirm that the following items are present in the figure legend, table legend, main text, or Methods section.

n/a	Confirmed	
☐	☒	The exact sample size (*n*) for each experimental group/condition, given as a discrete number and unit of measurement
☐	☒	A statement on whether measurements were taken from distinct samples or whether the same sample was measured repeatedly
☐	☒	The statistical test(s) used AND whether they are one- or two-sided<br>*Only common tests should be described solely by name; describe more complex techniques in the Methods section.*
☐	☒	A description of all covariates tested
☐	☒	A description of any assumptions or corrections, such as tests of normality and adjustment for multiple comparisons
☐	☒	A full description of the statistical parameters including central tendency (e.g. means) or other basic estimates (e.g. regression coefficient) AND variation (e.g. standard deviation) or associated estimates of uncertainty (e.g. confidence intervals)
☐	☒	For null hypothesis testing, the test statistic (e.g. *F*, *t*, *r*) with confidence intervals, effect sizes, degrees of freedom and *P* value noted<br>*Give P values as exact values whenever suitable.*
☒	☐	For Bayesian analysis, information on the choice of priors and Markov chain Monte Carlo settings
☒	☐	For hierarchical and complex designs, identification of the appropriate level for tests and full reporting of outcomes
☒	☐	Estimates of effect sizes (e.g. Cohen's *d*, Pearson's *r*), indicating how they were calculated

Our web collection on statistics for biologists contains articles on many of the points above.

Software and code

Policy information about availability of computer code

Data collection	No software used during data collection.
Data analysis	Comparisons among three or more groups were performed by one-way analysis of variance and the L.S.D. test using SAS (v9.21, Cary, USA). Comparisons between two groups were performed by two-sided student's t-test using SAS. Data related with flow cytometry were analyzed by FlowJo software (VX, Tree Star). Differential expression of RNA sequencing data were analyzed by R software 4.1.1 with packages of edgeR (3.42.2). Western blotting data were quantified by ImageJ (1.54d, National Institutes of Health, Bethesda, MD). The crystal structures of proteins were modeled using Modeller (v9.18). Point-mutated protein structures were modeled by Pymol (v2.3). Molecular dynamics analysis was performed on the mutated models using Amber 16. Protein-molecule docking was analyzed by AutoDock (v4.2.6).

For manuscripts utilizing custom algorithms or software that are central to the research but not yet described in published literature, software must be made available to editors and reviewers. We strongly encourage code deposition in a community repository (e.g. GitHub). See the Nature Portfolio guidelines for submitting code & software for further information.

Data

Policy information about availability of data

All manuscripts must include a data availability statement. This statement should provide the following information, where applicable:

- Accession codes, unique identifiers, or web links for publicly available datasets
- A description of any restrictions on data availability
- For clinical datasets or third party data, please ensure that the statement adheres to our policy

All relevant data that support the findings of this work were available in a publicly accessible repository. All the databases/datasets used in the study were along with appropriately accessible links/accession-codes in the manuscript under the “Data availability” section as well as in this reporting summary. Protein crystal structure of human carboxylesterase in complex with Coenzyme A can be obtained from the Protein Data Bank (PDB ID: 2H7C). The crystal structure of CES1 was obtained from the Protein Data Bank (PDB ID: 3K9B). The RNA sequencing data were deposited in NCBI database with accession number PRJNA640462.

Research involving human participants, their data, or biological material

Policy information about studies with human participants or human data. See also policy information about sex, gender (identity/presentation), and sexual orientation and race, ethnicity and racism.

Reporting on sex and gender	n/a
Reporting on race, ethnicity, or other socially relevant groupings	n/a
Population characteristics	n/a
Recruitment	n/a
Ethics oversight	n/a

Note that full information on the approval of the study protocol must also be provided in the manuscript.

Field-specific reporting

Please select the one below that is the best fit for your research. If you are not sure, read the appropriate sections before making your selection.

☒ Life sciences ☐ Behavioural & social sciences ☐ Ecological, evolutionary & environmental sciences

For a reference copy of the document with all sections, see nature.com/documents/nr-reporting-summary-flat.pdf

Life sciences study design

All studies must disclose on these points even when the disclosure is negative.

Sample size	No sample-size calculation was performed. The sample sizes were chosen according to studies in a similar research area. These studies provided insights into the expected effect sizes and variances, which helped in determining the sample sizes that would be sufficient to detect meaningful differences or relationships. Although sample size calculations were not explicitly performed, efforts were made to ensure an adequate statistical power for the experiments. The sample sizes were chosen to achieve a balance between practical feasibility and the ability to detect significant effects or relationships with a reasonable level of confidence.
Data exclusions	No data were excluded from the analyses.
Replication	In terms of replication, we conducted experiments at > 3 times to ensure the reliability and robustness of the results. All replication attempts were successful.
Randomization	All samples, cells and mice were allocated randomly into experimental groups.
Blinding	In our study, investigators were not blinded to sample identities. The decision not to blind investigators to sample identities was based on the nature of the study and the specific experimental design. Blinding is commonly employed in research to minimize bias and ensure objectivity by preventing knowledge of sample identities from influencing the data acquisition or processing. However, in certain types of studies, blinding may not be feasible or necessary. In our case, the nature of the experimental setup or the specific techniques used might not have allowed for blinding of investigators. For example, in studies involving direct interactions with samples, such as cell culture experiments or histological analysis, blinding may not be practical as the investigators need to know the sample identities to accurately perform the procedures and interpret the results. Furthermore, blinding may not be necessary if objective and standardized methods were employed for data acquisition and processing. In our study, we ensured the use of standardized protocols and established criteria for data collection and analysis. This helped to minimize potential bias and maintain consistency across the study.

Reporting for specific materials, systems and methods

We require information from authors about some types of materials, experimental systems and methods used in many studies. Here, indicate whether each material, system or method listed is relevant to your study. If you are not sure if a list item applies to your research, read the appropriate section before selecting a response.

Materials & experimental systems

n/a	Involved in the study
☐	☒ Antibodies
☐	☒ Eukaryotic cell lines
☒	☐ Palaeontology and archaeology
☐	☒ Animals and other organisms
☒	☐ Clinical data
☒	☐ Dual use research of concern
☒	☐ Plants

Methods

n/a	Involved in the study
☒	☐ ChIP-seq
☐	☒ Flow cytometry
☒	☐ MRI-based neuroimaging

Antibodies

Antibodies used

1. PE Rat Anti-Mouse CD3, Abcam, Ab22268;
2. APC Rat Anti-Mouse CD19, Abcam, Ab25484;
3. Alexa Fluor 488 Rat Anti-Mouse CD31, Bio-Rad, MCA2388A488T;
4. Brilliant Violet 785 Rat Anti-Mouse CD68, BioLegend, 137035;
5. DAPI, Becton Dickinson, 564907;
6. PE Rat IgG2a Isotype Control, Abcam, Ab253047;
7. APC Rat IgG2a Isotype Control, Abcam, Ab239461;
8. Alexa Fluor 488 Rat IgG2a Isotype control, Bio-Rad, MCA1212A488;
9. Brilliant Violet 785 Rat IgG2aκ Isotype control, BioLegend, 400274;
10. Nrf2, Cell Signaling Technology, 12721;
11. Ces2, Abcam, Ab215042.

Validation

1. Ab22268: Suitable for: Flow Cyt; Reacts with: Mouse; Verified with mouse spleen cells by Flow Cytometry analysis; cited by Liu C et al. Nat Nanotechnol 17:531-540 (2022); Website: https://www.abcam.cn/products/primary-antibodies/pe-cd3-antibody-kt3-ab22268.html;
2. Ab25484: Suitable for: Flow Cyt; Reacts with: Mouse; Verified with mouse splenocytes by Flow Cytometry analysis; cited by Harding CL et al. Front Cell Infect Microbiol 10:328 (2020), Krop I et al. Eur J Immunol 26:238-42 (1996), Krop I et al. J Immunol 157:48-56 (1996), Fearon DTs. Curr Opin Immunol 5:341-8 (1993);Website: https://www.abcam.cn/products/primary-antibodies/apc-cd19-antibody-6d5-ab25484.html;
3. MCA2388A488T: Suitable for: Flow Cyt; Reacts with: Mouse; Verified with murine peripheral blood; cited by Sumagin, R. & Sarelius, I. H. J Immunol 184(9):5242-52; Website: https://www.bio-rad-antibodies.com/monoclonal/mouse-cd31-antibody-er-mp12-mca2388.html?f=purified&utm_source=citeab.com&utm_medium=referral&utm_campaign=3rd+party+directory;
4. 137035: Suitable for: Flow Cyt; Reacts with: Mouse; Verified with mouse macrophages by Flow Cytometry analysis; cited by Kayama H, et al. 2012. PNAS. 109:5010, Park S, et al. 2013. Biomaterials. 34:598, Guiducci C, et al. 2013. J Exp Med; Website: https://www.biolegend.com/en-us/products/brilliant-violet-785-anti-mouse-cd68-antibody-19346;
5. 564907: Suitable for: Flow Cyt; Reacts with: Mouse; Verified with mouse pancreas by immunofluorescence analysis; cited by Darzynkiewicz Z, et al. Cytometry. 1992 13(8):795-808, Hotz MA, et al. Cytometry. 1994 15(3):237-244, Otto F. Methods Cell Biol. 1990 33:105-110; Website: https://www.bdbiosciences.com/en-us/products/reagents/flow-cytometry-reagents/research-reagents/single-colorantibodies-
ruo/dapi-solution.564907;
6. Ab253047: Suitable for: Flow Cyt; TargetSpecies: Negative Control; Website: https://www.abcam.cn/products/primary-antibodies/pe-rat-igg2a-monoclonal-2a3-isotype-control-ab253047.html;
7. Ab239461: Suitable for: Flow Cyt; TargetSpecies: Negative Control; Website: https://www.abcam.cn/products/primary-antibodies/apc-mouse-igg2a-monoclonal-mopc-173-isotype-controlab239461.html;
8. MCA1212A488: Suitable for: Flow Cyt; TargetSpecies: Negative Control; Verified with mouse splenocytes by Flow Cytometry analysis; cited by Guo C et al. Int J Med Mushrooms. 2011;13:237-44; Website: https://www.labome.com/product/Bio-Rad/MCA1212A488.html;
9. 400274: Suitable for: Flow Cyt; TargetSpecies: Negative Control; Verified with mouse macrophages by Flow Cytometry analysis; cited by Cao Y, et al. 2019. Nat Commun. 10:1280; https://www.biolegend.com/en-us/products/brilliant-violet-785-mouse-igg2a-kappa-isotype-ctrl-8959;
10. 12721: Suitable for: ChIP, WB; Reacts with: Mouse; Verified with MEF by WB analysis and ChIP analysis; cited by 529 papers such as Eric Grignano, et. al. Cell Death Discov. 2023 17;9(1):97; Website: https://www.cellsignal.com/products/primary-antibodies/nrf2-d1z9c-xp-rabbit-mab/12721;
11. Ab215042: Suitable for: WB; Reacts with: Mouse; Verified with mouse liver tissue by WB analysis; cited by Li H et al. Front Pharmacol 13:843872 (2022); Website: https://www.abcam.com/products/primary-antibodies/ces2-antibody-ab215042.html.

Eukaryotic cell lines

Policy information about cell lines and Sex and Gender in Research

Cell line source(s)	CHO, LO2, NCTC1459
Authentication	LO2 cell line was obtained from Cell Bank of Chinese Academy of Sciences (Shanghai, China). NCTC1469 was obtained from Tongpai Shanghai Biological Technology (Shanghai, China). CHO cells were obtained from Puhebio (Jiangsu, China). Both cell lines were authenticated by STR profiling.
Mycoplasma contamination	All cell lines were tested for mycoplasma contamination. No mycoplasma contamination was found.
Commonly misidentified lines (See ICLAC register)	No commonly misidentified cell lines were used in the study.

Animals and other research organisms

Policy information about studies involving animals; ARRIVE guidelines recommended for reporting animal research, and Sex and Gender in Research

Laboratory animals	Wild-type mice, db/db mice, Nrf2-deficient (Nrf2-/-) mice, and Ces2h-deficient (Ces2h-/-) mice were used for animal experiments. Wild-type mice, and db/db mice were obtained fromModel Animal Research Center of Nanjing University. Nrf2-/- mice, and Ces2h-/- mice were produced using the CRISPR-CAS9 system. All mice had a C57BL/6 genetic background. Male mice with 8 weeks were used.
Wild animals	The study did not involve wild animals.
Reporting on sex	Male mice were used to exclude the gender effect.
Field-collected samples	The study did not involve samples collected from the field.
Ethics oversight	All animal experiments were approved by the Animal Use and Care Committee of Zhejiang University (Hangzhou, China).

Note that full information on the approval of the study protocol must also be provided in the manuscript.

Flow Cytometry

Plots

Confirm that:

☒ The axis labels state the marker and fluorochrome used (e.g. CD4-FITC).

☒ The axis scales are clearly visible. Include numbers along axes only for bottom left plot of group (a 'group' is an analysis of identical markers).

☒ All plots are contour plots with outliers or pseudocolor plots.

☒ A numerical value for number of cells or percentage (with statistics) is provided.

Methodology

Sample preparation	The viability of the isolated cells was checked using 4',6-diamidino-2-phenylindole (DAPF) and incubated with fluorescent-labelled antibodies, which are cell surface antibodies including anti-CD3-PE, anti-CD19-APC, anti-CD31-Alexa Fluor 488, anti-Cd58-Brilliant Violet 785 (Table 51). After being stained under dark conditions at 4°C for 30 minutes, cells were washed twice in phosphate buffer saline (PBS), fixed and permeated in BD Cytofix/Cytoperm (BD Biosciences), and analyzed by a Flow Cytometer (FACSVerse: Becton Dickinson, Franklin Lakes, NJ, USA).
Instrument	Flow Cytometer (FACSVerse; Becton Dickinson, Franklin Lakes, NJ, USA)
Software	FlowJo software (VX, Tree Star)
Cell population abundance	The representative cell population was provided in the supplementary materials (Fig.S6-S7).

Gating strategy	The gating strategies were presented in the supplementary materials (Fig.S7). Respective isotype controls were used to standardize the background fluorescence (Table S1). The singlets were chosen for cells with similar area and height in forward scatter (FSC-A vs FSC-H). Nanoparticle-positive cells were measured by their fluorescence (Cy5.5, excitation/ emission: 675/694 nm) channel, with negative control set as nanoparticle-untreated, fully surface stained samples. Data were analyzed by FlowJo software (VX, Tree Star). The percentage of nanoparticle- positive cells was defined as the percentage of interested cells that fall within the nanoparticle-positive gate. Mean fluorescence intensity (MFI) represented the amount of nanoparticles absorbed by each cell Relative MFI was obtained by normalizing MFI to the control animals to account for inter-animal variability.

☒ Tick this box to confirm that a figure exemplifying the gating strategy is provided in the Supplementary Information.
